# Reverse Genetic Assessment of the Roles Played by the Spike Protein and ORF3 in Porcine Epidemic Diarrhea Virus Pathogenicity

**DOI:** 10.1128/jvi.01964-22

**Published:** 2023-06-26

**Authors:** Claudia Kristen-Burmann, Peter Rogger, Inês Berenguer Veiga, Stefanie Riebesehl, Julie Rappe, Nadine Ebert, Carmen A. Sautter, Jenna N. Kelly, Hanspeter Stalder, Rosina Ehmann, Michael Huber, Horst Posthaus, Nicolas Ruggli, Volker Thiel, Gergely Tekes

**Affiliations:** a Institute of Virology, Justus Liebig University Giessen, Germany; b Institute of Virology and Immunology (IVI), Bern and Mittelhäusern, Switzerland; c Department of Infectious Diseases and Pathobiology, Vetsuisse Faculty, University of Bern, Switzerland; d Institute of Animal Pathology, Vetsuisse-Faculty, University of Bern, Switzerland; e Institute of Medical Virology, University of Zürich, Switzerland; f COMPATH, University of Bern, Switzerland; Loyola University Chicago - Health Sciences Campus

**Keywords:** porcine epidemic diarrhea virus, synthesized DNA, vaccinia virus reverse genetics, spike gene, ORF3

## Abstract

Porcine epidemic diarrhea virus is a swine pathogen that has been responsible for significant animal and economic losses worldwide in recent years. In this manuscript, we report the generation of a reverse genetics system C(RGS) for the highly virulent US PEDV strain Minnesota (PEDV-MN; GenBank accession number KF468752), which was based on the assembly and cloning of synthetic DNA, using vaccinia virus as a cloning vector. Viral rescue was only possible following the substitution of 2 nucleotides within the 5′UTR and 2 additional nucleotides within the spike gene, based on the sequence of the cell culture-adapted strains. Besides displaying a highly pathogenic phenotype in newborn piglets, in comparison with the parental virus, the rescued recombinant PEDV-MN was used to confirm that the PEDV spike gene has an important role in PEDV virulence and that the impact of an intact PEDV ORF3 on viral pathogenicity is modest. Moreover, a chimeric virus with a TGEV spike gene in the PEDV backbone generated with RGS was able to replicate efficiently *in vivo* and could be readily transmitted between piglets. Although this chimeric virus did not cause severe disease upon the initial infection of piglets, there was evidence of increasing pathogenicity upon transmission to contact piglets. The RGS described in this study constitutes a powerful tool with which to study PEDV pathogenesis and can be used to generate vaccines against porcine enteric coronaviruses.

**IMPORTANCE** PEDV is a swine pathogen that is responsible for significant animal and economic losses worldwide. Highly pathogenic variants can lead to a mortality rate of up to 100% in newborn piglets. The generation of a reverse genetics system for a highly virulent PEDV strain originating from the United States is an important step in phenotypically characterizing PEDV. The synthetic PEDV mirrored the authentic isolate and displayed a highly pathogenic phenotype in newborn piglets. With this system, it was possible to characterize potential viral virulence factors. Our data revealed that an accessory gene (ORF3) has a limited impact on pathogenicity. However, as it is also now known for many coronaviruses, the PEDV spike gene is one of the main determinants of pathogenicity. Finally, we show that the spike gene of another porcine coronavirus, namely, TGEV, can be accommodated in the PEDV genome background, suggesting that similar viruses can emerge in the field via recombination.

## INTRODUCTION

Porcine epidemic diarrhea virus (PEDV) is an important pathogen of swine that causes high economic losses worldwide (1). This virus has been responsible for the death of over seven million piglets in the United States alone, since its introduction in 2013 (2). The causative agent of porcine epidemic diarrhea (PED) is a member of the family *Coronaviridae*, order *Nidovirales*. Within the subfamily *Orthocoronavirinae*, PEDV belongs to the genus *Alphacoronavirus* (3). Besides a close relationship to the human coronaviruses 229E (HCoV-229E) and NL63 (HCoV-NL63), PEDV is more distantly related to transmissible gastroenteritis virus (TGEV), which is another important *Alphacoronavirus* that infects swine and is clinically indistinguishable from PEDV (3). Like other coronaviruses, PEDV is enveloped and contains a single-stranded, positive-sense RNA genome of 28 kb that encodes seven open reading frames (ORFs). The 5′ two-thirds of the genome encodes two large polyproteins, namely, pp1a and pp1ab, that are processed into 16 nonstructural proteins to form the replication and transcription machinery (4, 5). The 3′ 8 kb of the genome encodes four structural genes, namely, the spike (S), envelope (E), membrane (M), and nucleocapsid (N), as well as one accessory gene (6–9). Since the S glycoprotein is the main inducer of neutralizing antibodies (10) and promotes the entry of virions into cells as well as cell-to-cell fusion (11), it plays a pivotal role in the virulence of different PEDV strains as well as in viral growth *in vitro* (12–15). Whereas the N-terminal S1 domain that is responsible for receptor binding is prone to mutations (16), the C-terminal fusion domain is more conserved (15). The requirement of proteolytic cleavage for the activation of the S protein has hampered the *in vitro* propagation of PEDV for many years, until Hofmann and Wyler were able to propagate the virus in the presence of trypsin in 1988 (17). To date, the isolation and cell culture propagation of PEDV field strains still remains challenging (18, 19). The accessory protein 3, which is encoded by ORF3 downstream of the S gene, exhibits ion channel activity and modulates virus production (20). Furthermore, it is dispensable for the growth of PEDV in cell culture (21), since in many cell culture adapted strains, ORF3 is disrupted, resulting in a truncated protein (22). ORF3-defective mutants have already been used as vaccines because they show less virulence in the field, but further studies applying reverse genetics are required to investigate the correlation between ORF3 and virulence in animal experiments.

After the infection of intestinal epithelial cells, PEDV results in severe diarrhea, vomiting, and dehydration in pigs, leading to high mortality rates between 80 and 100% in newborn piglets (2, 23). In older pigs, infection with PEDV causes less severe disease, possibly due to the faster turnover rates of enterocytes (1). Nevertheless, the infection of feeder and fattening pigs seems to be important for the endemic maintenance of PEDV in swine farms (24).

After the first outbreak of PED in the United Kingdom in 1971, the prototype strain CV777, originating from Belgium in 1978 (25), was adapted to cell culture. After several PED outbreaks all over Europe during the 1980s and 1990s, the incidence of cases in European countries decreased, and PEDV became endemic in Asia, where highly virulent strains that differed genetically from the previously isolated viruses evolved since 2010 (23, 26). Fatality rates of up to 100% in newborn piglets and the emergence of PED in vaccinated herds was attributed to insertions and deletions, especially in the S gene, which rendered pigs that were vaccinated with a CV777-based vaccine partially susceptible to PEDV (23). In 2013, the first outbreak of PED in the United States was observed in Iowa, and the highly virulent variants quickly spread all over the country and to Canada and Central America. To date, 39 states in the US have been affected by the disease, which kills nearly 10% of the US pig population. Although the exact route of introduction of PEDV into the US remains unknown, sequence analyses showed a close relationship to Asian strains (16, 27). Lately, besides low virulent strains (28, 29), highly virulent PEDVs that are related to the US variants were detected in some European countries (30). In addition to highly virulent strains, there is also evidence for the circulation of S-INDEL strains (31), which are characterized by insertions and deletions in the S1 domain, compared to highly virulent strains. In most cases, these variants seem to be associated with less virulence in the field and might have evolved from recombination events involving a Chinese strain (32). Notably, the discovery of chimeric porcine coronaviruses with a TGEV backbone and a PEDV spike gene in Italy and Germany demonstrates that natural recombination is possible between PEDV and TEGV (33, 34).

Reverse genetic systems (RGS) are powerful tools with which to study the molecular pathogenesis of PED and the genomic differences that lead to low or high virulence, as they allow for the introduction of well-defined mutations into the viral genome and for the evaluation of their impact on disease development *in vivo*. To date, several reverse genetic platforms have been reported for PEDV. In 2013, Li et al. described the first RGS for the cell-adapted DR13 vaccine strain, proving that ORF3 is not essential for replication *in vitro* (21). However, the targeted RNA recombination system that was used in this study only allows the manipulation of approximately 8 kb at the 3′ end of the genome (35). The generation of infectious cDNA coronavirus clones via the bacterial artificial chromosome (BAC) system was achieved for strain AVCT12 (36) as well as for two recent Chinese field isolates (37, 38). For the US field isolates, only one RGS, based on *in vitro* ligation, has been reported so far (39).

Here, we report the generation of a RGS for the highly virulent US PEDV strain Minnesota (PEDV-MN; GenBank accession number KF468752), based on the assembly and cloning of synthetic DNA, using vaccinia virus as a cloning vector. This technology allows for the stable propagation of full-length coronaviral cDNA and enables the genetic modification of the cDNA by vaccinia virus-mediated homologous recombination (5, 40–44). Although the cloned full-length cDNA exactly matched the PEDV-MN sequence that was deposited into GenBank, the rescue of a recombinant virus (recPEDV-MN) was only possible after the substitutions of 2 nucleotides within the 5′UTR and of 2 additional nucleotides within the spike gene, based on the sequence of the cell culture-adapted CV777 strain. With these modifications, the recombinant PEDV-MN displayed a highly pathogenic phenotype in newborn piglets, demonstrating that this RGS provides a powerful tool with which to study PEDV pathogenesis. Furthermore, we have applied this system to show that the impact of an intact ORF3 on viral pathogenicity is modest, and we confirmed that the PEDV spike gene is an important viral determinant for pathogenicity. Moreover, we demonstrate that a chimeric virus with the TGEV spike gene in the PEDV backbone is viable, replicates efficiently *in vivo*, and can be readily transmitted between piglets. Notably, although this chimeric virus did not cause severe disease upon the initial infection of piglets, we observed evidence of increasing pathogenicity after transmission to contact piglets.

## RESULTS

### Generation of a synthetic full-length PEDV DNA clone.

**FIG 1:**
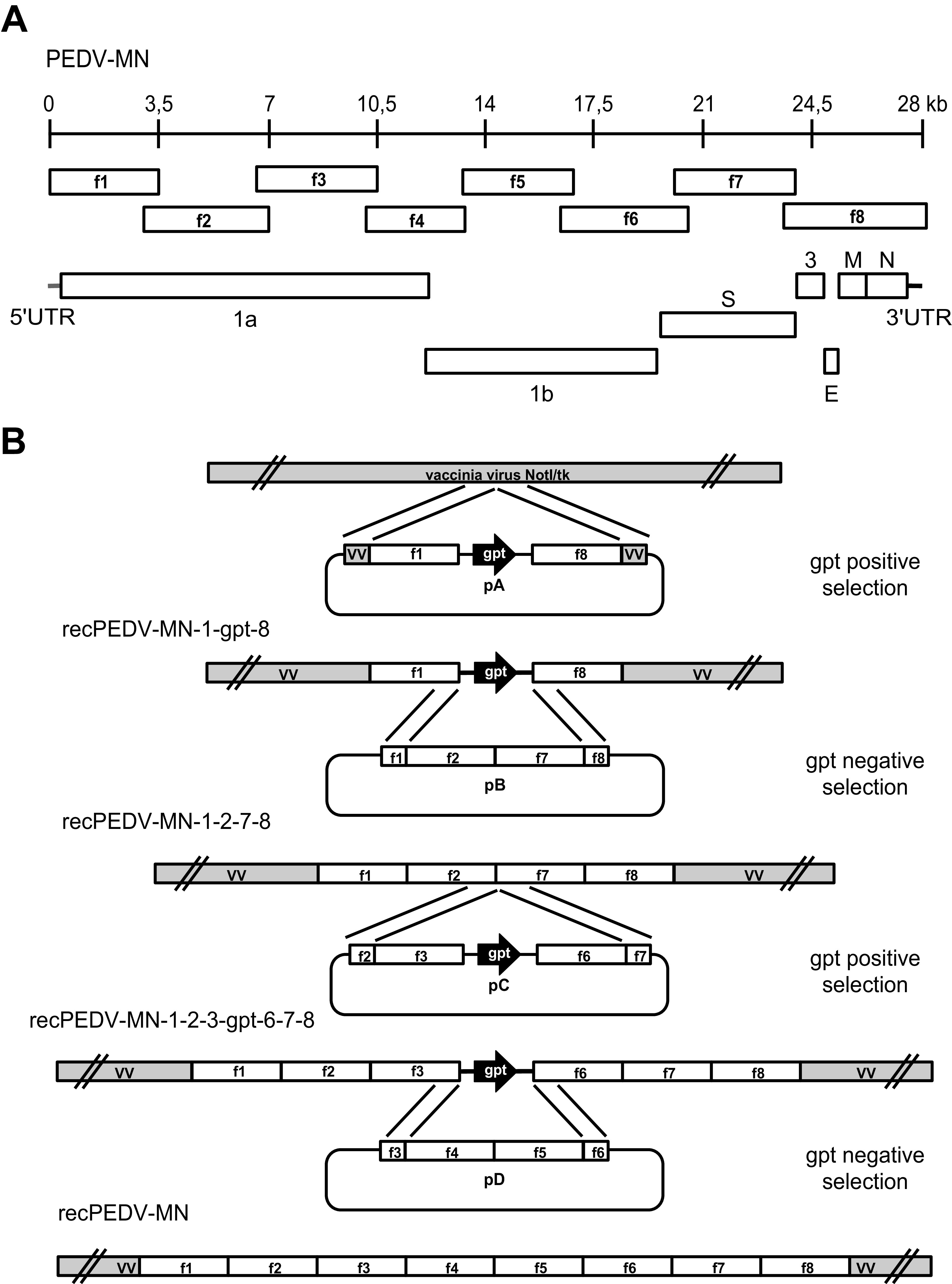
Schematic diagram of PEDV genome organization and strategy for the generation of RGS of a highly virulent US PEDV strain (PEDV-MN). (A) The +ssRNA genome of PEDV is represented schematically with a 28 kb scale, the 8 overlapping genome fragments 1 to 8 (f1 to f8) used for cloning the viral cDNA, the 5′ and 3′UTRs, and the ORFs, namely, 1a and 1b, as well as the accessory gene 3 (ORF3), the spike (S), envelope (E), membrane protein (M), and nucleocapsid (N) protein genes. (B) Eight plasmids (p1 to p8) expressing the eight overlapping synthetic cDNA fragments f1 to f8, encompassing the whole PEDV-MN genome, were generated. Subsequently, these plasmids were used to generate four plasmids within the pGPT-1 plasmid backbone, namely, pA, which encoded fragments 1 and 8 upstream and downstream of the *gpt* gene, respectively; pB, which encoded fragments 2 and 7; pC, which contained fragments 3 and 6 upstream and downstream of the *gpt* gene, respectively; and pD, which consisted of fragment 5 subcloned into the p4 backbone. These plasmids were then used to introduce the full-length PEDV-MN cDNA into the vaccinia virus NotI/tk backbone in four rounds of vaccinia virus-mediated double recombination, using GPT alternatively as a positive/negative selection marker. These rounds led to the generation of three intermediate constructs, namely, recPEDV-MN-1-gpt-8, recPEDV-MN-1-2-7-8, recPEDV-MN-1-2-3-gpt-6-7-8, and, ultimately, recPEDV-MN.

Eight cDNA fragments corresponding to the full-length genomic sequence of a highly pathogenic PEDV strain isolated in Minnesota (PEDV-MN; GenBank accession number KF468752) (16) were synthesized. A full-length PEDV-MN cDNA copy (originally recPEDV-MN) was assembled via our vaccinia virus-based RGS (5, 40–44) (Fig. 1). The original recPEDV-MN RNA was obtained following linearization and *in vitro* transcription, and it was used for viral rescue after electroporation in BHK-PEDV_MN_-N, as previously described (36–39). Surprisingly, and despite repeated attempts, no recombinant PEDV could be recovered.

### The rescue of recombinant PEDV required changes in the 5′UTR and the spike gene.

**FIG 2:**
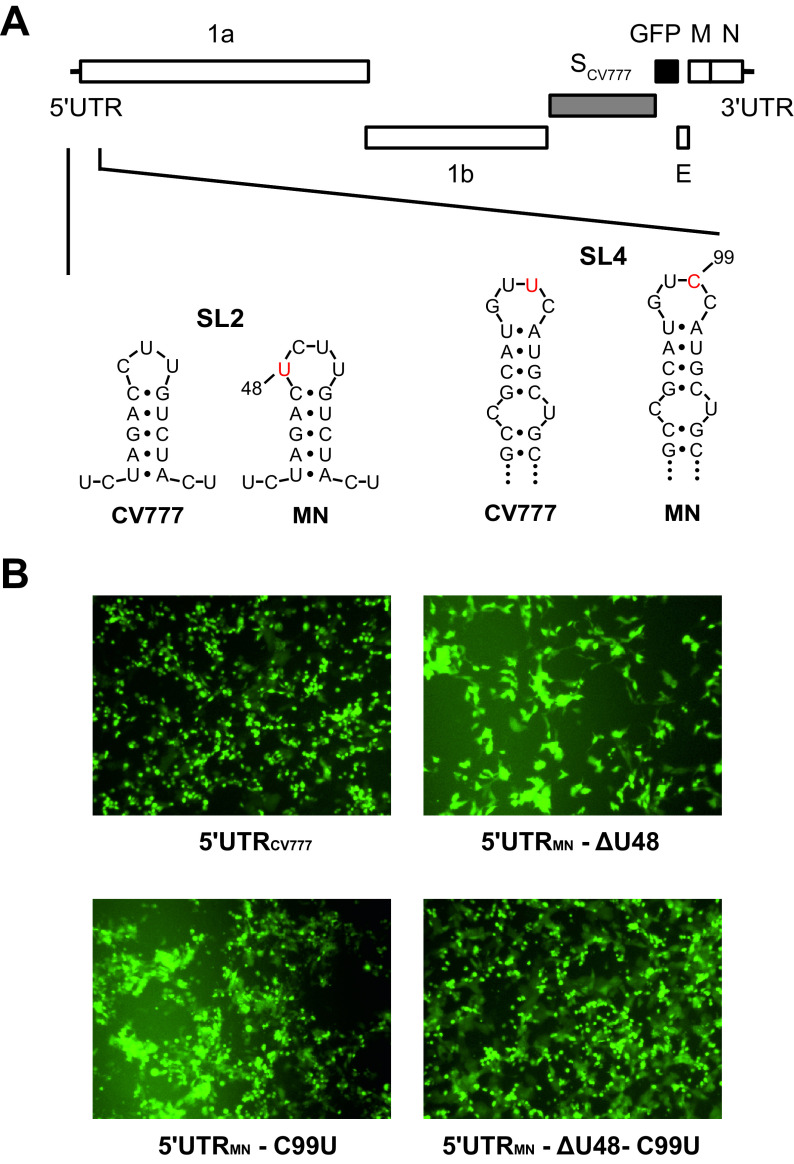
The rescue of recombinant PEDV-MN-S_CV777_-ΔORF3-GFP required the repair of the 5′-UTR region. (A) The sequence analysis of 5′UTR_MN_ and 5′UTR_CV777_ via RACE-PCR disclosed four nucleotide (nt) differences. According to an RNA secondary structure prediction, two of these nucleotides (nt 48 and 99) were located at the tip of stem-loop 2 and stem-loop 4 (SL2 and SL4), respectively. (B) GFP expression was associated with the successful rescue of recombinant viruses containing alterations of the 5′UTR_MN,_ namely, recPEDV-MN-5′UTR_CV777_-S_CV777_-ΔORF3-GFP (upper left, 5′UTR_CV777_), recPEDV-MN-5′UTR_MN-ΔU48_-S_CV777_-ΔORF3-GFP (upper right, 5′UTR_MN_-ΔU48), recPEDV-MN-5′UTR_MN-C99U_-S_CV777_-ΔORF3-GFP (lower left, 5′UTR_MN_-C99U), and recPEDV-5′UTR_MN-ΔU48, C99U_-S_CV777_-ΔORF3-GFP (lower right, 5′UTR_MN_-ΔU48-C99U).

It is known that the isolation and cell culture propagation of PEDV field strains are challenging tasks and that the PEDV S protein is a key determinant for virus growth *in vitro* (12–15, 17–19). Since no information on the *in vitro* propagation of strain MN was available in the literature, we sought to elucidate the reason for the unsuccessful rescue. To enable the recovery and propagation of recombinant viruses, we decided to modify the PEDV strain MN infectious clone. We replaced the S gene with the corresponding gene of the cell culture adapted prototype strain CV777. Furthermore, the ORF3 gene was replaced by a GFP-encoding gene, resulting in recPEDV-MN-S_CV777_-ΔORF3-GFP (Fig. 2A), to facilitate the detection of recovered viruses and to exclude that the full-length ORF3 may preclude successful rescue (36). However, despite these modifications, we were still unable to recover any recombinant PEDVs from recPEDV-MN-S_CV777_-ΔORF3-GFP, suggesting that regions other than the S and gene 3 of the PEDV genome hampered the recovery of recombinant PEDVs. Given the importance of conserved RNA structures in the CoV 5′UTR for replication, transcription, and translation (16, 45–48), we investigated whether the 5′UTR_MN_ was responsible for the unsuccessful rescues. Since the PEDV strain CV777 grows well in cell culture, we determined its 5′UTR sequence via 5′RACE-PCR from viral RNA that originated from cells that were infected with our CV777 laboratory strain. Notably, we observed two differences between our CV777 strain and the published sequence (GenBank accession number AF353511), namely, that the published sequence has an insertion of an A nucleotide at position 72 and an insertion of 4 nucleotides (UCCU) at positions 86 to 89 (according to the nucleotide numbering in the published AF353511 sequence). Therefore, we replaced the complete 5′UTR of recPEDV-MN-S_CV777_-ΔORF3-GFP with the 5′UTR_CV777_ of our laboratory CV777 strain via vaccinia virus-mediated homologous recombination. Following the *in vitro* transcription of recPEDV-MN-5′UTR_CV777_-S_CV777_-ΔORF3-GFP DNA, the RNA was electroporated into BHK-PEDV_MN_-N cells. After 48 h of incubation, the supernatant was harvested and used to infect Vero cells, as described previously. At 24 h postinfection (p.i.), GFP-expressing Vero cells were identified via fluorescence microscopy, and, upon the addition of trypsin to the growth medium, a pronounced cytopathic effect developed, indicating the successful rescue of recombinant PEDVs (Fig. 2B) (5′UTR_CV777_). The identity of the recovered recombinant PEDV (recPEDV-MN-5′UTR_CV777_-S_CV777_-ΔORF3-GFP) that originated from the infected Vero cells was confirmed via a full-length Sanger sequence analysis. These results demonstrate that the exchange of the 5′UTR_MN_ for the 5′UTR_CV777_ enabled the efficient recovery of recombinant PEDVs.

A sequence comparison of the 5′UTR_MN_ and the 5′UTR_CV777_ of our CV777 laboratory strain revealed four nucleotide (nt) differences (Fig. 2A). At nt position 48, the strain MN contained one additional nt (U), and, at nt 99, the strain MN contained a C instead of a U (T99C), compared to CV777. Furthermore, whereas CV777 contained a G at nt 117 and a C at nt 287, the strain MN possessed an A (G117A) and a U (C287U) at these two positions, respectively. An RNA secondary structure prediction of the 5′UTR indicated that nt 48 and nt 99 are located at the tip of stem-loop 2 (SL2) and SL4, respectively (Fig. 2A) (16). Since the G117A and C287U modifications did not affect the predicted RNA structure of the PEDV 5′UTR, we focused on nt 48 and 99. Accordingly, three recombinant vaccinia viruses that contained the 5′UTR_MN_ with alterations were generated: (i) in recPEDV-MN-5′UTR_MN-ΔU48_-S_CV777_-ΔORF3-GFP, the U at position 48 was deleted (ΔU48); (ii) in recPEDV-MN-5′UTR_MN-C99U_-S_CV777_-ΔORF3-GFP, the C at position 99 was replaced with a U (C99U); and (iii) recPEDV-MN-5′UTR_MN-ΔU48,C99U_-S_CV777_-ΔORF3-GFP contained both modifications (ΔU48 and C99U). After rescue, recombinant PEDVs from all three infectious clones could be recovered, resulting in recPEDV-MN-5′UTR_MN-ΔU48_-S_CV777_-ΔORF3-GFP, recPEDV-MN-5′UTR_MN-C99U_-S_CV777_-ΔORF3-GFP, and recPEDV-MN-5′UTR_MN-ΔU48,C99U_-S_CV777_-ΔORF3-GFP, respectively (Fig. 2B). The identities of these recombinant PEDVs were confirmed via a full-length Sanger sequence analysis following the performance of RT-PCR. All three viruses replicated efficiently in Vero cells, showed GFP expression, displayed the same plaque morphology, and reached high titers that were comparable to the titer of recPEDV-MN-5′UTR_CV777_-S_CV777_-Δ3-GFP that contained the entire 5′UTR_CV777_. These experiments demonstrated that the deletion or exchange of one single nucleotide on the tip of SL2 or SL4 enabled the efficient recovery of recombinant PEDVs.

### Effect of the ORF3 gene on the recovery of recombinant PEDVs.

**FIG 3:**
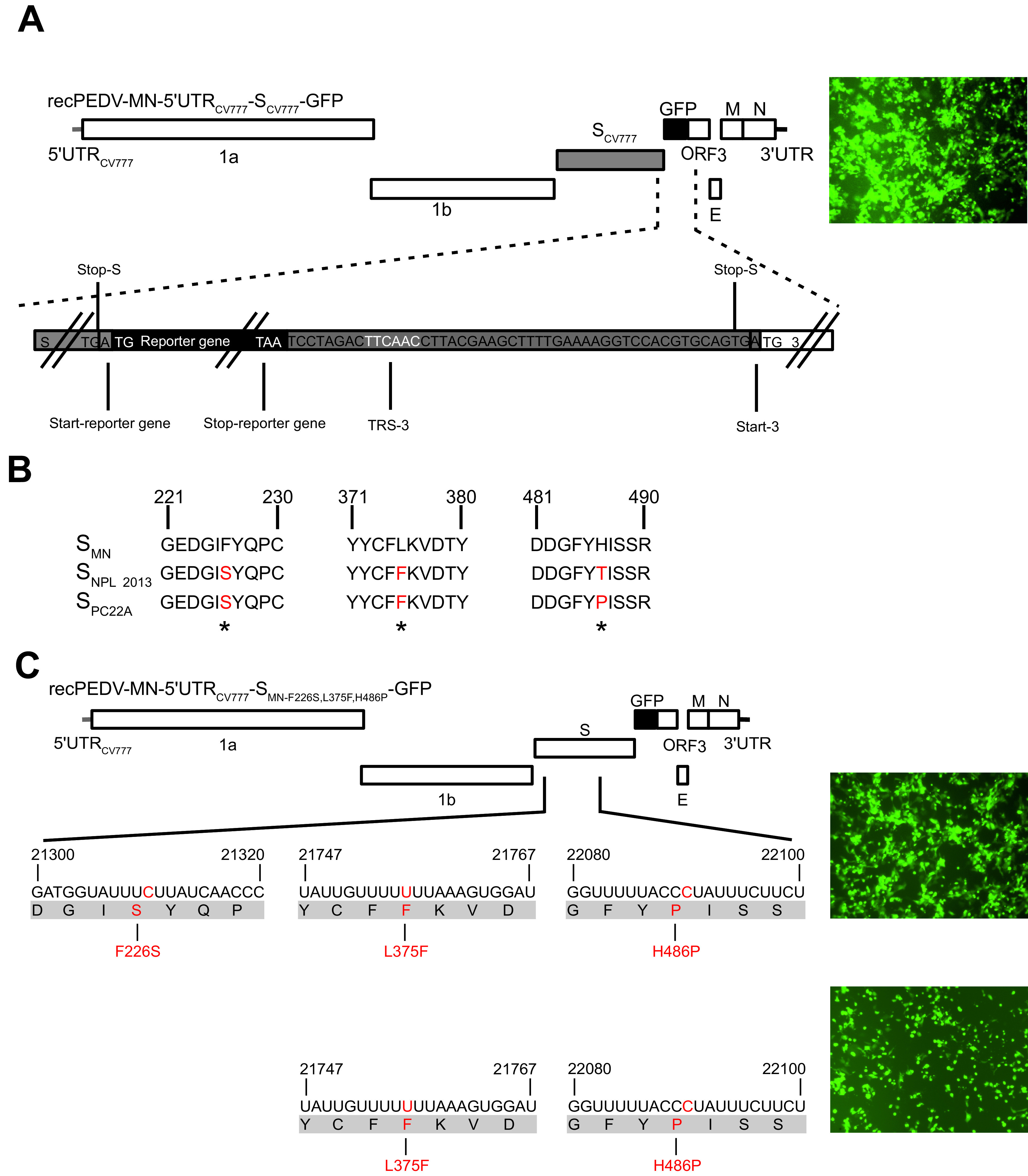
The presence or absence of the ORF3 gene does not significantly impair the recovery of recombinant PEDVs. The sequence analysis and correction of the PEDV-MN spike gene enabled PEDV-MN rescue. (A) Assembly of recPEDV-MN-5′UTR_CV777_-S_CV777_-GFP. The last 49 nucleotides from the S gene that include the TRS from ORF3 were inserted downstream of the GFP reporter gene to enable the expression of the ORF3 gene. The GFP expression was associated with the successful rescue of this recombinant virus, as shown on the right. (B) The alignment of selected portions of the amino acid sequence of PEDV-S_MN_ with the corresponding sequences of two highly virulent field strains (PEDV-NPL 2013, PEDV-PC22A) revealed three differences in the receptor binding domain (S1) which are highlighted in red. (C) The replacement of the amino acid at positions 226, 376, and 486 in S_MN_ with the corresponding residues of S_PC22A_ resulted in a green fluorescence signal, indicating the rescue of recPEDV-MN-5′UTR_CV777_-S_MN-F226S, L375F, H486P_-GFP. The phenylalanine at position 375 and the proline at 486 (recPEDV-MN-5′UTR_CV777_-S_MN-L375F, H486P_-GFP) are essential for virus rescue.

Jengarn and coworkers failed to rescue recombinant PEDV with an intact ORF3 from a cDNA clone (36). Accordingly, it was suggested that an intact ORF3 was able to suppress PEDV replication *in vitro*. To revisit this hypothesis, we reintroduced an intact ORF3 gene into recPEDV-MN-5′UTR_CV777_-S_CV777_-ΔORF3-GFP between the GFP and E genes, resulting in recPEDV-MN-5′UTR_CV777_-S_CV777_-GFP. The recombinant PEDVs (recPEDV-MN-5′UTR_CV777_-S_CV777_-GFP) could be recovered (Fig. 3A), and the identity of the virus was determined via a full-length Sanger sequence analysis. Taken together, this result did not confirm the previous hypothesis that an intact ORF3 gene suppresses viral replication in cell culture, and we were able to recover recombinant PEDVs with the same efficiency, independent of the presence or absence of an intact ORF3 gene.

### Changes in the PEDV-MN spike gene enabled the rescue of recombinant PEDV-MN.

Since we showed that the efficient recovery of recombinant PEDVs was possible with an intact ORF3 gene and the replacement of the 5′UTR_MN_ with that of strain CV777, we asked whether the S protein of strain MN instead of S_CV777_ can also support the rescue and propagation of recombinant viruses *in vitro*. Accordingly, in recPEDV-MN-5′UTR_CV777_-S_CV777_-GFP, the S gene of strain CV777 was replaced by the corresponding part of strain MN with vaccinia virus-mediated homologous recombination, resulting in recPEDV-MN-5′UTR_CV777_-S_MN_-GFP. Surprisingly, we were not able to recover the recombinant PEDV, and no GFP expression or cytopathic effect could be observed in Vero cells that were infected with the cell culture supernatant of electroporated BHK-PEDV_MN_-N cells. Therefore, we compared the amino acid sequence of S_MN_ with the S protein sequences of two highly virulent field strains that also grew in cell culture, namely, PEDV-NPL 2013 (GenBank accession number KM052365) and PEDV-PC22A (GenBank accession number KM392224) (18, 40, 49–51) (Fig. 3B).

The sequence analyses revealed four and five amino acid differences in the receptor-binding domain (S1) of S_MN_, compared to S_NPL2013_ and S_PC22A_, respectively. Interestingly, three of these residues were different in S_MN_, compared to both S_PC22A_ and S_NPL2013_. At position 226, S_MN_ contained phenylalanine, whereas both S_NPL2013_ and S_PC22A_ possessed serine. In S_MN,_ aa 376 was a leucine residue, whereas the other two S proteins had phenylalanine at this position. The third amino acid at position 486 was different in all three strains (histidine in S_MN_, threonine in S_NPL2013_, and proline in S_PC22A_). We decided to replace these three amino acids at positions 226, 376, and 486 in S_MN_ with the corresponding residues of S_PC22A,_ resulting in recPEDV-MN-5′UTR_CV777_-S_MN-F226S,L375F,H486P_-GFP. Vero cells that were infected with the electroporation supernatant displayed green fluorescence and syncytium formation (Fig. 3C), indicating the successful rescue of recPEDV-MN-5′ UTR_CV777_-S_MN-F226S,L375F,H486P_-GFP with three modifications in the S_MN_. To narrow down which residue(s) out of these three enabled the rescue of recombinant PEDVs, a panel of recombinant vaccinia viruses was generated (data not shown). Our analyses revealed that phenylalanine at 375 and proline at 486 were indispensable for virus rescue and growth *in vitro*, whereas the presence of serine or phenylalanine at 226 did not influence virus recovery or cell culture propagation. The identity of recPEDV-MN-5′UTR_CV777_-S_MN-L375F,H486P_-GFP was confirmed via a Sanger sequence analysis of RT-PCR products from the mutated regions.

Based on these data, we completed the generation of an infectious PEDV-MN clone by adapting the synthetic PEDV-MN cDNA that was initially cloned, based on the sequence deposited on GenBank (KF468752), with the two changes in the 5′-UTR (the deletion of U48 [ΔU48] and the replacement of C99 with U) and the two changes in the spike gene sequence to encode a phenylalanine at aa position 375 and a proline at aa position 486. The resulting vaccinia virus containing the adapted PEDV-MN cDNA was used to rescue the recombinant PEDV-MN (recPEDV-MN), and its sequence was deposited into GenBank (OL704806).

### Synthetic recPEDV-MN is highly pathogenic in piglets. The impact of the spike gene.

To assess the pathogenicity of rec-PEDV-MN and to assess the impact of the PEDV spike gene on the pathogenicity, we constructed the recombinant recPEDV-MN-S_CV777_ with the spike gene of the laboratory strain PEDV-CV777 in the backbone of PEDV-MN and compared the phenotype with recPEDV-MN *in vivo*. For both recombinant viruses (recPEDV-MN and recPEDV-MN-S_CV777_), the supernatants of BHK-PEDV_MN_-N cells that had been electroporated with the respective RNAs were collected and termed passage 0 (P0). The P0 virus stocks were then used to determine the titers on Vero cells as well as the identities of the viruses. Notably, both P0 virus stocks contained an intact ORF3 gene and differed only in the spike gene.

Four groups of six piglets (6 to 9 days old) were infected orally with 10^4^ PFU of PEDV-CV777 (control for low pathogenic virus), PEDV-NLP2013 (control for high pathogenic virus), recPEDV-MN-S_CV777_, and recPEDV-MN, respectively. In the groups infected with PEDV-CV777 and recPEDV-MN-S_CV777_, one piglet out of six piglets died at 1 day postinfection due to non-PEDV-related causes. The remaining five piglets from these groups were included in downstream analyses. Four piglets were mock-infected with DMEM and served as controls. The piglets were monitored for clinical signs for seven days. In addition, at day 2 p.i., two naive contact piglets that had previously received infectious virus were added to each group to assess virus transmission. The mock piglets were euthanized four days after the infection of the remaining groups.

**FIG 4:**
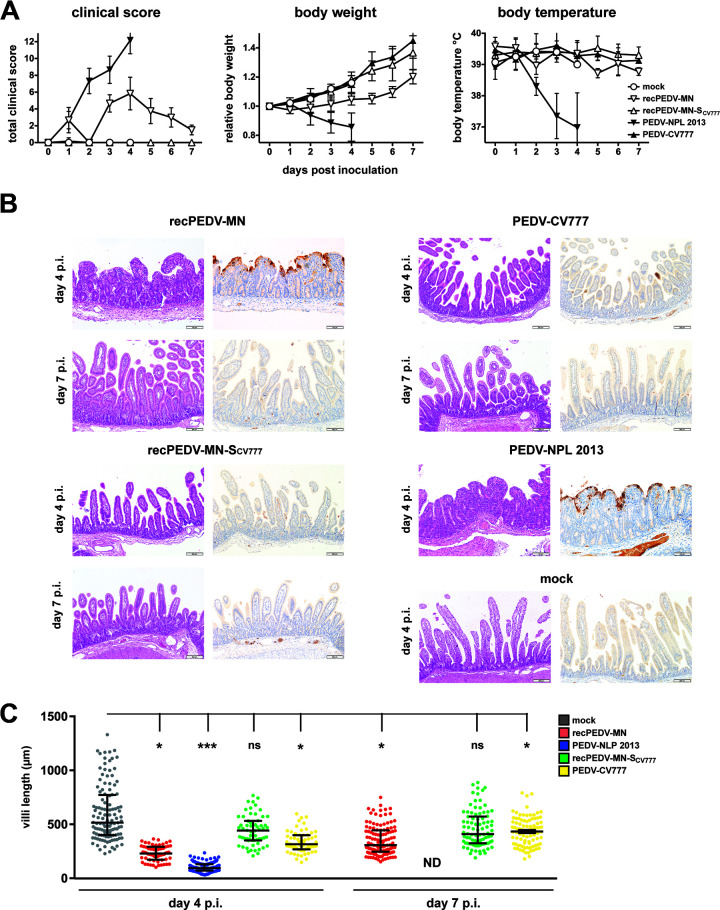
Synthetic recPEDV-MN is highly pathogenic in piglets, and the spike gene plays an important role in PEDV pathogenesis. (A) Clinical scores, body weights, and body temperatures of newborn piglets that were infected with the indicated PEDV isolates and recombinant viruses (recPEDV-MN [*n* = 6], recPEDV-MN-SCV777 [*n* = 5], PEDV-NPL 2013 [*n* = 6], PEDV-CV777 [*n* = 5]), compared with those of mock-infected piglets (*n* = 4) until 7 day p.i. Each dot represents a mean value, with error bars showing the standard deviation. (B) Histological and immunohistochemical findings in the jejunum of a representative piglet each at days 4 and 7 p.i., with the indicated viruses shown in comparison to a mock-infected piglet. H&E staining is shown on the left, and anti-PEDV_M_ IHC is shown on the right. Longer bar, 200 μm; shorter bar, 100 μm. (C) Comparison of the jejunal villous length displayed by each of the six piglets from the four groups on days 4 (left) and 7 (right) p.i., in comparison with mock-infected piglets on day 4 p.i. The data were analyzed via a multilevel linear regression model (*P* < 0.05). One asterisk (*) stands for *P* < 0.05, three asterisks (***) stand for *P* < 0.001, and ns stands for a nonsignificant statistical difference.

**(i) Clinical signs.** Severe clinical signs, such as vomitus, watery diarrhea, and loss of appetite were observed as soon as 12 h p.i. in piglets that were infected with PEDV-NPL 2013 and recPEDV-MN. The severity of clinical signs increased dramatically in the piglets of both groups and included severe dehydration, among other signs. The total clinical scores of the piglets that were infected with PEDV-NPL 2013 reached the discontinuation criteria on day 4 p.i. (Fig. 4A). However, although the piglets that were infected with recPEDV-MN also showed severe clinical signs, the discontinuation criteria were not reached, and the piglets improved after the peak of the infection (Fig. 4A). Notably, the contact piglets that were added to the PEDV-NPL 2013-infected and recPEDV-MN-infected piglets on day 2 p.i. also displayed severe clinical signs as soon as 12 h after cohousing. In sharp contrast, neither the piglets that were infected with PEDV-CV777 or recPEDV-MN-S_CV777_ nor the corresponding contact piglets showed any clinical signs of disease (Fig. 4A).

**(ii) Pathological and immunohistochemical findings.** Consistent with the clinical signs that were observed, the small and large intestines of the piglets that were infected with PEDV-NPL 2013, as well as those of the respective contact piglets, contained a large amount of gas and watery, transparent stool mixed that was with yellowish and greenish flocculated material and few nondigested vegetable fibers (data not shown). Additionally, the stomachs were severely distended and filled with a large amount of watery, whitish fluid, and some animals displayed focal acute mucosal hemorrhages in the cardial and cranial fundic regions. Histologically, there was a severe atrophy and fusion of the small intestinal villi with multifocal villus tip necrosis as well as a multifocal to a coalescing, strongly positive cytoplasmic and mostly apical membranous signal in the anti-PEDV IHC (Fig. 4B; Fig. S1A). The pathological and immunohistochemical findings were similar in piglets that were infected with recPEDV-MN on day 4 p.i. (Fig. 4B) and in the respective contact piglets after 2 days of cohousing (Fig. S1A). However, the intestinal contents of the PEDV-MN-infected piglets became indistinguishable from those of the mock-infected piglets on day 7 p.i. (data not shown). Histologically, these animals displayed normally appearing intestinal villi and a marked decrease in PEDV viral antigen detection, compared to those observed on day 4 p.i. (Fig. 4B). The piglets infected with PEDV-CV777 and recPEDV-MN-S_CV777_ as well as the respective contact piglets remained like the mock piglets throughout the experiment. However, there was a statistically significant difference in intestinal villi length in the group that was infected with PEDV-CV777, compared with the mock-infected piglets on both days 4 and 7 p.i. (Fig. 4C) (multilevel linear regression model, *P* < 0.05). This effect was less pronounced than were the effects that were observed for PEDV-NPL 2013 and recPEDV-MN for the same time points.

**FIG 5:**
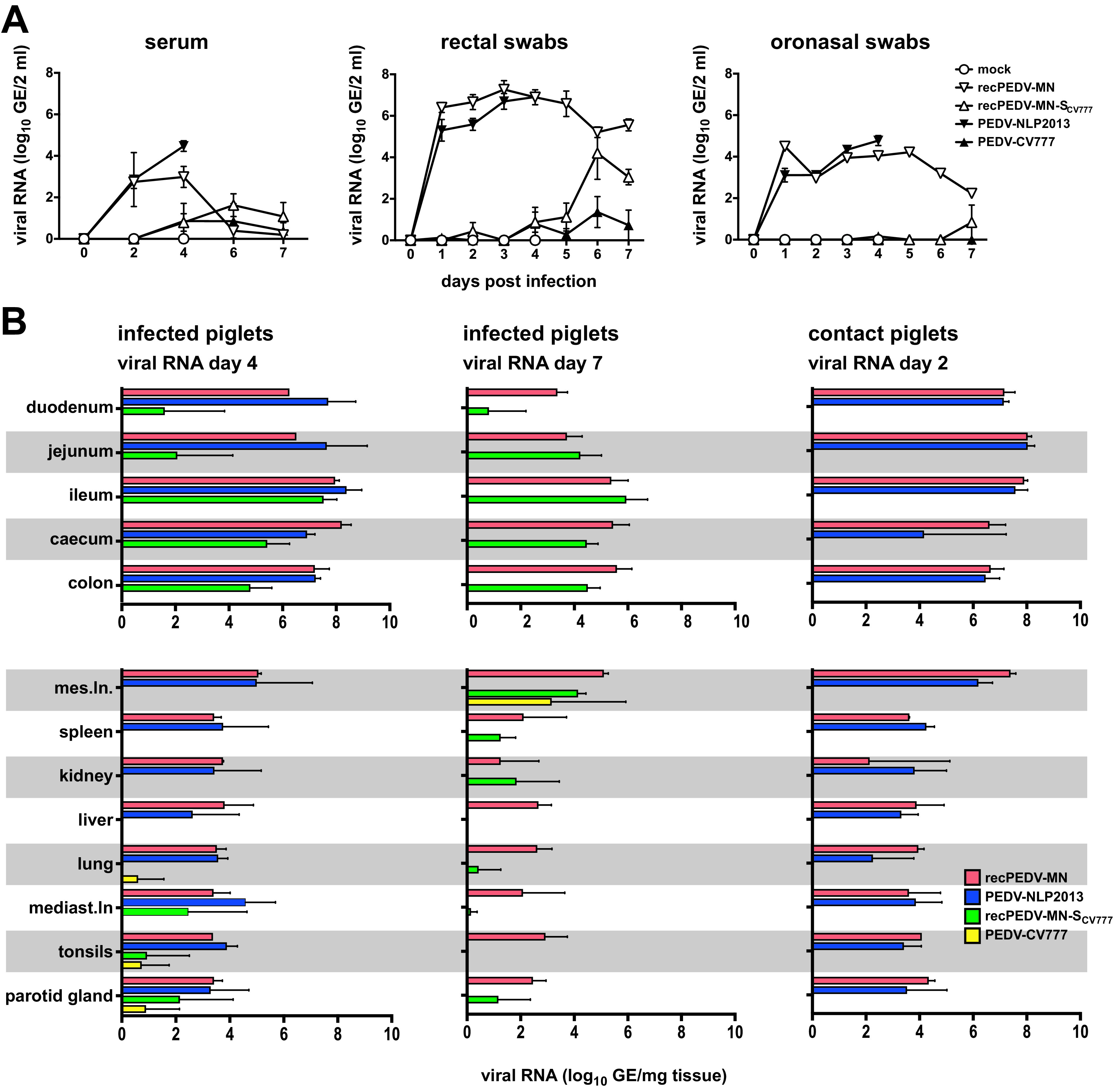
RNA shedding and RNA distribution in the tissues in the piglets that were infected with synthetic recPEDV-MN resemble the values displayed by PEDV-NPL 2013. (A) Viral RNA shedding in serum, rectal, and oronasal swabs of piglets that were either infected with the indicated viruses or mock-infected. (B) Viral RNA loads in log_10_ genome equivalents (GE) per mg of tissue samples from primary infected or contact piglets from the four groups that were euthanized at the indicated days p.i. Each dot (panel A) or bar (panel B) represents a mean value, with error bars showing the standard deviation.

**(iii) Viral RNA.** Viral RNA in serum and fecal and oronasal swabs could be detected in samples from all of the infected piglets; however, in accordance with clinical and pathological examinations, the viral RNA loads were highest in the samples that were derived from PEDV-NPL 2013-infected and recPEDV-MN-infected piglets (Fig. 5A). Notably, we observed that viral RNA loads in the feces and serum of recPEDV-MN-S_CV777_-infected and PEDV-CV777-infected piglets slightly increased, starting from day 4 p.i., and they decreased on day 7 p.i. Viral RNA was detectable in all of the tissue samples that were derived from the PEDV-NPL 2013-infected and recPEDV-MN-infected piglets on day 4 p.i., and particularly high viral RNA loads were observed in the intestinal tissues (Fig. 5B). In contrast, infection with the two low pathogenic viruses recPEDV-MN-S_CV777_ and PEDV-CV777 resulted in generally lower viral RNA loads in tissue samples. However, in samples derived from the ileum, cecum, and colon, the recPEDV-MN-S_CV777_ viral RNA loads reached values that were close to those of PEDV-NPL 2013 and recPEDV-MN on day 4 p.i., and, on day 7 p.i., the viral RNA loads of recPEDV-MN-S_CV777_ were comparable to those of recPEDV-MN in all of the intestinal samples, except for the duodenum samples (Fig. 5B). This suggests that recPEDV-MN-S_CV777_ retained considerable ability to replicate *in vivo*, whereas PEDV-CV777 RNA was detectable in only a few tissue samples, suggesting that PEDV-CV777 is more severely attenuated *in vivo* than is recPEDV-MN-S_CV777_.

**(iv) Virus transmission.** As mentioned above, the contact piglets of the PEDV-NPL 2013-infected and recPEDV-MN-infected piglets displayed severe clinical PED as soon as 12 h after cohousing, whereas the contact piglets of the recPEDV-MN-S_CV777_-infected and PEDV-CV777-infected piglets did not show any clinical signs. Accordingly, viral RNA was detected in all tissues in the samples that were derived from the contact piglets of PEDV-NPL 2013-infected and recPEDV-MN-infected piglets, suggesting that the transmission of PEDV-NPL 2013 and recPEDV-MN is highly efficient (Fig. 5B). In contrast, we could not detect viral RNA in the contact piglets of recPEDV-MN-S_CV777_-infected and PEDV-CV777-infected piglets. However, it should be noted that cohousing was only performed for 2 days (i.e., from day 2 to day 4 p.i.) and that the viral RNA in the feces of recPEDV-MN-S_CV777_-infected and PEDV-CV777-infected piglets remained low until day 5 p.i., after which, toward the end of the experiment, higher viral RNA loads were observed. Therefore, we cannot exclude that the transmission of recPEDV-MN-S_CV777_ or PEDV-CV777 may occur at later time points p.i.

Collectively, the assessment of clinical symptoms, pathology and immunohistochemistry, viral RNA loads, and viral transmission revealed that the recombinant virus recPEDV-MN displays a highly pathogenic phenotype, whereas the recombinant virus recPEDV-MN-S_CV777_, in which we have exchanged the PEDV-MN spike gene for the spike gene of the laboratory strain PEDV-CV777, has a low pathogenic phenotype.

### The ORF3 gene has a minor impact on PEDV-MN pathogenicity.

**FIG 6:**
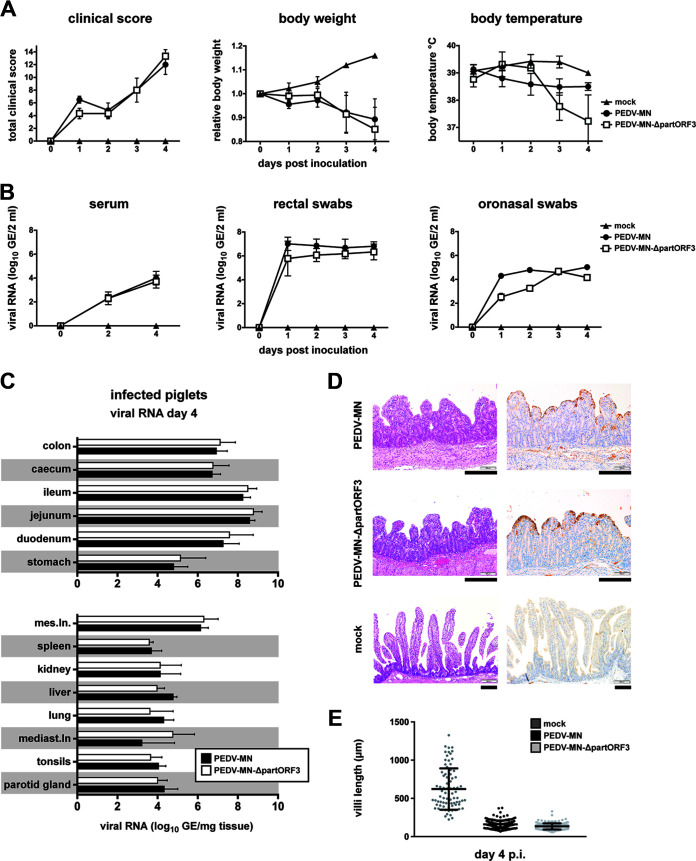
The ORF3 gene plays a minor role in PEDV pathogenesis. (A) Clinical scores, body weights and body temperatures of the piglets (*n* = 6 per group) that were infected with 10^4^ PFU recPEDV-MN-ΔpartORF3 and parental recPEDV-MN, in comparison with mock-infected piglets. (B) Daily viral RNA in log_10_ GE per 2 mL in serum, rectal, and oronasal swabs of piglets that were infected with recPEDV-MN-ΔpartORF3 and recPEDV-MN, in comparison with mock-infected piglets. (A) Viral RNA load in log_10_ GE per mg tissue detected in organ samples from piglets on day 4 p.i. with recPEDV-MN-ΔpartORF3 and recPEDV-MN. Each dot (panels A and B) or bar (panel C) represents a mean value, with error bars showing the standard deviation. (D) Histological and immunohistochemical findings in the jejunum of a representative piglet per group. H&E staining is shown on the left, and anti-PEDVM IHC is shown on the right. Longer bar, 200 μm; shorter bar, 100 μm. (E) The comparison of villi length displayed at day 4 p.i. by piglets that were infected with both viruses, in comparison with mock-infected piglets.

Several coronavirus accessory genes have been described as pathogenicity factors, and, for some of these, defined functions, for example, in the context of innate immune evasion, have been elucidated (52–54). PEDV contains one accessory gene, namely, the ORF3 gene, that encodes a tetrameric ion channel protein (20). Notably, upon the propagation of PEDV field strains in tissue culture, the ORF3 gene is rapidly disrupted by small deletions, resulting in truncated versions of the ORF3 (22; our observation). This finding gave rise to speculation regarding whether the ORF3 gene may impact PEDV pathogenicity. Therefore, we addressed the question of whether a deletion within the ORF3 impacts PEDV pathogenicity. For this purpose, we introduced a deletion of 49 nucleotides in the ORF3 of recPEDV-MN that mimics the disrupted ORF3 of our laboratory strain PEDV-CV777. The deletion of 49 nucleotides in the ORF3 of our PEDV-CV777 is located 123 nucleotides downstream of the ORF3 start codon (41 codons, including the start codon), and it causes a frameshift that allows for the translation of an additional 10 residues before a stop codon terminates translation. This truncated ORF3 version contains only the aminoterminal part of ORF3. This is less than one-third of the full-length ORF3 protein, and it is considered to be nonfunctional, at least for the ion channel activity. We compared the phenotype of the resulting recombinant virus recPEDV-MN-ΔpartORF3 with that of the parental recPEDV-MN. Two groups of six piglets each were infected orally with 10^4^ PFU of recPEDV-MN and recPEDV-MN-ΔpartORF3, respectively. Clinical signs of severe PED became apparent as soon as 12 h p.i. in animals of both groups. Piglets that were infected with recPEDV-MN-ΔpartORF3 displayed lower body temperatures at day 4 p.i., but, otherwise, these animals were indistinguishable from the recPEDV-MN-infected piglets with respect to clinical signs, histopathological examination, and viral antigen and viral RNA loads (Fig. 6). Piglets in both groups reached the clinical score for termination of the experiment at day 4 p.i. (Fig. 6A), suggesting that both viruses are equally highly pathogenic in piglets, following oral infection with 10^4^ PFU. The recombinant virus recPEDV-MN-ΔpartORF3 was also readily transmissible, as two contact piglets that were added to the group of recPEDV-MN-ΔpartORF3-infected piglets only displayed clinical signs that were indistinguishable from those of the recPEDV-MN-inoculated or recPEDV-MN-ΔpartORF3-inoculated piglets (data not shown).

**FIG 7:**
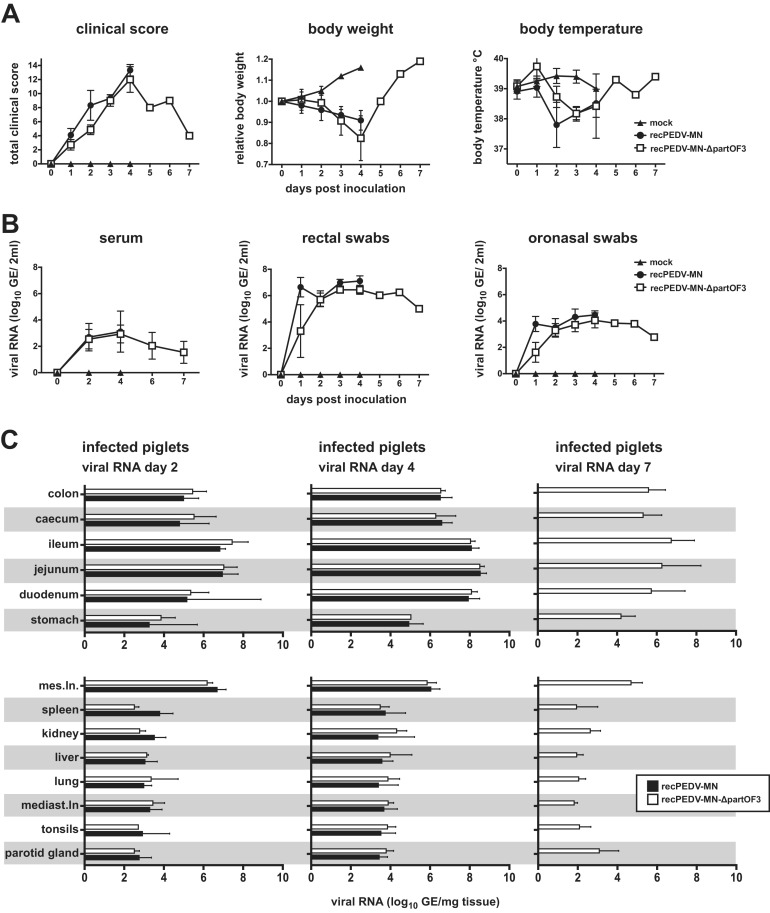
A lower dose of infection does not significantly impact the outcome of recPEDV-MN-ΔpartORF3 infection in newborn piglets. (A) Clinical scores, body weights, and body temperatures of the piglets infected with 10^3^ PFU recPEDV-MN-ΔpartORF3 and recPEDV-MN, in comparison with mock-infected piglets. (B) Daily viral RNA in GE per 2 mL in serum, rectal, and oronasal swabs of piglets that were infected with recPEDV-MN-ΔpartORF3 and recPEDV-MN, in comparison with mock-infected piglets. (C) Viral RNA load in log_10_ GE per mg tissue detected in organ samples from piglets on days 2, 4, and 7 p.i. with recPEDV-MN-ΔpartORF3 and recPEDV-MN. Each dot (panels A and B) or bar (panel C) represents a mean value, with error bars showing the standard deviation. (D) Histological and immunohistochemical findings in the jejunum of a representative piglet per group. H&E staining is shown on the left, and anti-PEDV_M_ IHC is shown on the right. Longer bar, 200 μm; shorter bar, 100 μm. (E) Comparison of villi length displayed at day 4 p.i. by piglets infected with both viruses, in comparison with mock-infected piglets.

We repeated this experiment with a lower dose of infection (10^3^ PFU; orally) to assess whether, under these conditions, the difference in the ORF3 gene between recPEDV-MN and recPEDV-MN-ΔpartORF3 may impact pathogenicity. For this *in vivo* experiment, nine piglets were added to each group, and three piglets were euthanized on days 2, 4, and 7 p.i. Again, we did not observe any major differences concerning the time point of the onset of clinical signs of disease (at 12 h p.i. in both groups), clinical scores, or viral RNA loads in the feces, oronasal swabs, serum, or other various tissues within the first 4 days p.i. (Fig. 7). However, on day 4 p.i., the recPEDV-MN-infected piglets displayed slightly higher total clinical scores that reached the discontinuation criteria, whereas the clinical scores of the recPEDV-MN-ΔpartORF3-infected piglets were just below the threshold for discontinuation (Fig. 7A). Interestingly, as observed for the recPEDV-MN-infected piglets in the first experiment, we noted that the clinical signs of the recPEDV-MN-ΔpartORF3-infected piglets improved rapidly after day 4, and this was concomitant with increasing body weight and decreasing viral RNA loads (Fig. 7). Also, while the piglets from all groups displayed severe villus atrophy and fusion on days 2 and 4 p.i., the piglets that were infected with recPEDV-MN-ΔpartORF3 displayed an increase in villi length at day 7 p.i., and this was associated with the loss of PEDV viral antigen detection (Fig. S2). Collectively, these data suggest that a truncated ORF3 gene has only a minor impact on viral pathogenicity.

### A PEDV/TGEV chimeric virus is replication competent and has the potential for increased pathogenicity.

**FIG 8:**
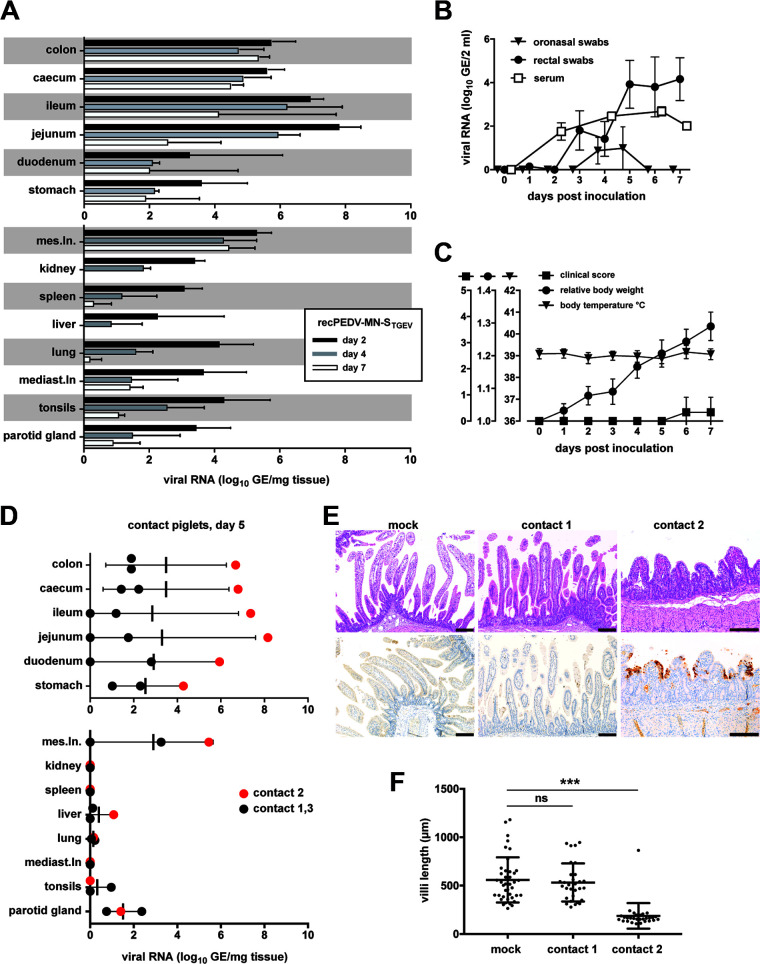
A chimeric PEDV/TGEV virus is viable *in vitro* and *in vivo*, and it has the potential for increased pathogenicity upon *in vivo* passaging. (A) A group of nine piglets was infected with recPEDV-MN-S_TGEV_ and cohoused with contact piglets (*n* = 3) at 2 days after infection. The viral RNA loads in log_10_ GE per mg tissue were determined in several tissue samples from infected piglets at days 2 (*n* = 3), 4 (*n* = 3), and 7 p.i. (*n* = 3). (B) Daily viral RNA loads in log_10_ GE per 2 mL serum, rectal, and oronasal swabs from infected piglets. (C) Clinical scores, body weights, and body temperatures of infected piglets. Each bar (panel A) or dot (panels B and C) represents a mean value, with error bars showing the standard deviation. (D) Viral RNA loads in log_10_ GE per mg tissue in the indicated contact piglets (*n* = 3) at day 5 p.i. (E) Histological and immunohistochemical findings in the jejunum of contact piglets 1 and 2 on day 4 post introduction, in comparison with a representative mock-infected piglet. H&E staining is shown in the top panels, and anti-PEDV_M_ IHC is shown in the bottom panels. Longer bar, 200 μm; shorter bar, 100 μm. (F) Comparison of villi length on day 4 post introduction of contact piglets 1 and 2, in comparison with mock-infected piglets. The data were analyzed via a Kruskal-Wallis H test (*P* < 0.05). Three asterisks (***) stand for *P* < 0.001, and ns stands for a nonsignificant statistical difference.

The recent detection of chimeric viruses consisting of a TGEV genomic backbone with a spike gene region that originated from PEDV in Europe demonstrated that TGEV and PEDV can recombine in the field (33, 34). This event raises concerns that novel recombinants with increased pathogenicity may arise. Therefore, we addressed the questions of whether chimeric viruses that are based on a genomic backbone of a highly pathogenic PEDV strain with a spike gene originating from TGEV Purdue (GenBank accession number DQ811789.2) would be replication competent and whether such viruses may have a highly pathogenic phenotype. Remarkably, we could readily rescue a recombinant virus recPEDV-MN-S_TGEV_ from cloned DNA that contained the TGEV Purdue spike gene in the PEDV-MN backbone. The chimeric virus grew to titers that were comparable to those of the parental recPEDV-MN, demonstrating that such recombinants are likely viable if they arise in the field. For the *in vivo* experiment, nine piglets were infected with the recPEDV-MN-S_TGEV_, and four piglets were infected with recPEDV-MN. Three piglets from the recPEDV-MN-S_TGEV_ were euthanized on days 2, 4 and 7 p.i, whereas two piglets were from the recPEDV-MN group were euthanized on days 2 and 4 p.i. Three contact piglets were added to the recPEDV-MN-S_TGEV_ group only at two days after infection. In this experiment, recPEDV-MN-S_TGEV_ was attenuated (Fig. 8), in comparison with recPEDV-MN (Fig. 4 and 6). Although the viral RNA loads were considerably high in the ileum and jejunum at 2 days p.i., they declined by several orders of magnitude until day 7 p.i. (Fig. 8A). Viral RNA was transiently detectable in oronasal swabs (days 4 and 5 p.i.) and was detectable in rectal swabs and in serum until the termination of the experiment (Fig. 8B). The clinical score remained low throughout the infection, and the piglets displayed normal body temperatures and gained weight (Fig. 8C). This suggests that this chimeric virus can efficiently replicate *in vivo* but has a low virulent phenotype. We also observed that the chimeric virus could transmit to all three contact piglets that were cohoused beginning at 2 days p.i. and analyzed for viral RNA loads in intestinal and nonintestinal tissues at day 5 post cohousing (Fig. 8D). The contact animals did not show any clinical signs of disease, with the exception that contact piglet 2 had a reduced appetite at day 4 post cohousing. However, we noted that the viral RNA loads were particularly high in the intestinal organs of this piglet. Upon histological examination, we identified the severe atrophy and fusion of the intestinal villi (Fig. 8E) as well as statistically significantly reduced villi length in this contact piglet, in comparison with the mock-infected piglets (Fig. 8F) (Kruskal-Wallis H test, *P* < 0.05), as was observed in previous experiments in piglets that were infected with highly pathogenic PEDV-NPL 2013 or recPEDV-MN. Therefore, we sequenced the viral RNA from the jejunum of this contact piglet and detected 6 nucleotide changes with frequencies of >10% within the PEDV-derived sequences of ORF1ab, E, M, and N as well as 4 changes in the TGEV Purdue-derived spike sequence (Table 1). Notably, at nucleotide position 21,584 of recPEDV-MN-S_TGEV_, we detected a U to C mutation with a frequency of 97.6%, resulting in a substitution of F to L at amino acid position 318 of the TGEV-Purdue spike protein. This substitution has already been detected among several amino acid changes that discriminate virulent (containing an L) from attenuated (containing an F) TGEV strain Miller. Unfortunately, the viral RNA loads in the organs of the other contact piglets were too low to obtain RT-PCR products for sequencing, but we were able to determine the sequence of one piglet that had initially been infected. The recPEDV-MN-S_TGEV_ sequence that was obtained from this animal also contained several nucleotide changes, but they were distinct from the nucleotide changes that were detected in the recPEDV-MN-S_TGEV_ sequence of contact piglet 2. This observation demonstrates that the nucleotide changes that were detected in recPEDV-MN-S_TGEV_-infected piglets and in contact piglet 2 arose during *in vivo* replication. Collectively, these results demonstrate that the TGEV spike protein is functional in the genomic PEDV background. The chimeric virus can readily replicate *in vitro* and *in vivo* and is efficiently transmitted to contact piglets. Importantly, the virus can rapidly acquire mutations *in vivo* and may have the potential to undergo a phenotypic change from an attenuated to a virulent phenotype upon passaging in pigs.

**TABLE 1:** Mutations identified following the sequencing of viral RNA recovered from contact piglet 2

Region	Position	Ref	Alt	Type	AF	Depth	AA
PEDV 1a	1,843	C	T	Missense	0.9731	1,745	5,18 R>C
PEDV 1a	3,638	C	T	Missense	0.4527	3,576	1,116 A>V
PEDV 1b	16,243	A	T	Missense	0.9984	5,143	1,215 H>L
Spike TGEV	21,584	T	C	Missense	0.9763	1,984	3,18 F>L
Spike TGEV	23,418	T	C	Missense	0.4441	8,153	9,29 I>T
Spike TGEV	23,891	C	A	Missense	0.4021	9,803	1,087 H>N
Spike TGEV	23,892	A	T	Missense	0.4063	9,730	1,087 H>L
E	25,920	A	C	Missense	0.5285	11,072	75 D>A
M	26,236	G	A	Missense	0.9987	8,519	101 R>Q
N	27,114	G	A	Missense	0.4453	4,821	163 S>N

## DISCUSSION

PEDV raised considerable concerns after the emergence of highly pathogenic variants in Asia and the outbreak in the US as well as in neighboring countries in 2013 (55). In response to this outbreak, we aimed to assess the pathogenicity of a contemporary highly pathogenic PEDV strain (PEDV-MN) and to raise preparedness, as similar highly pathogenic variants may reach Europe. To establish a reverse genetic system for highly pathogenic PEDV, we used an approach based on synthesized DNA fragments that we combined into a full-length cDNA clone using the vaccinia virus as a cloning vector. Therefore, although a recombinant highly pathogenic PEDV was eventually obtained, the original full-length PEDV-MN cDNA that was initially cloned based on a GenBank sequence turned out to not be viable. The identification of sequence variations within the PEDV 5′UTR and the spike gene was crucial to establish a reverse genetic system for the highly pathogenic PEDV-MN. In particular, it is known that conserved RNA structures in the CoV 5′UTR are important for replication, transcription, and translation (16, 45–48). Therefore, although the described differences at nt 48 and 99 do not alter the RNA structure of the 5′UTR, it is important to note that the number of nucleotides and the sequence conservation in SL2 and SL4 can be important for RNA-RNA or RNA-protein interactions and may consequently affect virus viability (45–48, 56). Our analysis over the entire genome did not reveal any other nucleotides within the original PEDV-MN sequence or the cloned recPEDV-MN genome that would not be present in other published PEDV sequences. Therefore, the reverse genetics system of recPEDV-MN is well-suited for use in the study of the determinants of PEDV pathogenicity.

Using this system, we have corroborated previous findings concerning the PEDV spike gene as an important factor for PEDV pathogenicity. By exchanging the PEDV-MN spike gene with the PEDV-CV777 spike gene, we observed that the resulting recPEDV-MN-S_CV777_ chimeric virus was able to replicate *in vitro* and *in vivo* but did not cause any noticeable signs of disease. However, recPEDV-MN-S_CV777_ showed robust replication, mainly in the intestinal organs of infected piglets, whereas PEDV-CV777 RNA was only detectable in some organs (Fig. 5B). This suggests that the PEDV-MN backbone contributes to pathogenicity. A similar observation has been made for mouse hepatitis virus (MHV), in which spike exchanges between the MHV strain A59 and the JHM strain showed a contribution of the backbone to neurovirulence (57).

In contrast to the spike gene, we did not observe any major contribution of the ORF3 to PEDV pathogenicity. It is known that the ORF3 gene of the cell-culture adapted strain PEDV-CV777 is interrupted by a small deletion, and it has been reported that the ORF3 gene is unstable upon the replication of PEDV in cell culture ([36]; our observation). Therefore, for our *in vivo* studies, we used passage 0 or passage 1 viruses throughout all experiments and carefully monitored the integrity of ORF3 in our virus stocks. By comparing the pathogenicity of recPEDV-MN (with intact ORF3) with recPEDV-MN-ΔpartORF3 (with interrupted ORF3), there was no noticeable difference in any recorded parameter (viral load, clinical score, body weight, body temperature, histology, immunohistochemistry) following the infection of piglets with 10^4^ PFU (Fig. 6). Even with a lower dose of infection (10^3^ PFU), most parameters were indistinguishable, and both viruses were readily transmitted to contact piglets. However, it should be noted that with this lower dose, the clinical score of piglets infected with recPEDV-MN reached the discontinuation criteria, whereas the clinical score of the recPEDV-MN-ΔpartORF3-infected piglets was just below the threshold for discontinuation (Fig. 7A). These results suggest that ORF3 is only of minor importance for PEDV pathogenicity. It was also noticeable that the recPEDV-MN-ΔpartORF3-infected piglets recovered rapidly from day 4 to day 7 p.i. (Fig. 7A), similar to the recPEDV-MN-infected piglet in our first experiment, in which the clinical score declined from day 4 to day 7 p.i. (Fig. 4A). This suggests that piglets that survive the peak of infection have a favorable prognosis to fully recover from the infection.

Coronaviruses are well known for their ability to recombine extensively. A classic example is the type II feline coronavirus (FCoV), which contains the genetic backbone of the type I FCoV and the spike region from canine coronavirus (58). Interestingly, there are several type II FCoVs that differ mainly in the exact regions of recombination within or adjacent to the spike gene, suggesting that recombination between feline and canine coronaviruses is not a rare event. Furthermore, extensive recombination can be observed for SARS-related bat coronaviruses (59) and, even more impressively, for various strains of avian infectious bronchitis virus (IBV) (60). The coronavirus spike protein is the major determinant for the elicitation of antibody responses, and it is often the spike gene that is affected by recombination in the field, suggesting that recombination is an important mechanism of immune escape for coronaviruses.

In accordance with the detection of chimeric porcine coronaviruses with a TGEV backbone and a PEDV spike gene that were described in Italy and Germany (33, 34), we were able to generate a chimeric virus with a PEDV backbone and a TGEV spike gene that was viable both *in vitro* and *in vivo* and was not associated with clinical and histopathological changes. Also, this chimeric virus was readily transmitted from piglet to piglet, and it was associated with the progression from an attenuated to a virulent phenotype that followed transmission in one contact piglet. These findings suggest that there may be a selection toward harmonization between the several viral components upon transmission with consequent increased viral pathogenicity. Interestingly, an artificially generated chimeric PEDV-TGEV virus has been shown to provide protection against virulent PEDV by replicating in the jejunum of infected piglets as efficiently as a virulent control virus in the absence of clinical and histopathological signs (61). However, since no contact piglets were used in this study, prior to our study, it remained unknown whether similar phenotypic changes could ensue after the transmission of this chimeric virus between piglets.

In conclusion, we report the successful generation of a RGS for the highly virulent US PEDV strain, Minnesota, which was shown to display a highly pathogenic phenotype in newborn piglets that was comparable to that of the parental virus. This system confirmed the important role that the PEDV spike gene plays in virulence and that the impact of an intact PEDV ORF3 on viral pathogenicity is modest. Furthermore, we could show that a chimeric virus with a PEDV backbone and a TGEV spike gene is viable, replicates efficiently *in vivo*, and can be readily transmitted from piglet to piglet. Although this chimeric virus did not cause severe disease upon the initial infection of the piglets, evidence of increasing pathogenicity after transmission to contact piglets was observed.

Therefore, the RGS generated here is a powerful tool in the study of PEDV pathogenesis and can be used in future research on PEDV as well as to develop vaccines against porcine enteric coronaviruses.

## MATERIALS AND METHODS

### Cells and viruses.

Baby hamster kidney cells (BHK-21) and African green monkey kidney cells (Vero DST) were originally purchased from the American Type Culture Collection (ATTC) and kindly provided by the diagnostic laboratory of our institute, respectively. The D980R cells were a kind gift from G. L. Smith, Imperial College, London, United Kingdom, and were used to generate the recombinant vaccinia viruses via homologous recombination. Monkey kidney (CV-1) cells were obtained from the European Collection of Cell Cultures. The BHK-Tet/ON cells were a kind gift from N. Tautz, University of Lübeck, Germany. All cells were maintained in minimal essential medium (MEM) supplemented with 10% fetal bovine serum, penicillin (100 U/mL), and streptomycin (100 U/mL). The PEDV strains CV777 (GenBank accession number AF353511) and NPL 2013 (GenBank accession number KM052365) were kindly provided by Mathias Ackermann, Institute of Virology, Vetsuisse Faculty, University of Zurich, Switzerland. The PEDVs were grown in Vero cells as follows. After 2 h postinoculation in serum free medium containing 80 μg/mL trypsin, cells were washed and maintained in MEM for 48 h. Subsequently, the medium was replaced with serum free MEM with trypsin (25 μg/mL) to enable the release of recombinant virus particles. The propagation, titration, and purification of recombinant vaccinia viruses were performed as described previously (5, 40, 41).

### Plasmid construction and generation of recombinant vaccinia viruses.

Based on the complete genome sequence of PEDV strain Minnesota (MN) deposited on GenBank accession number KF468752, eight plasmids (p1 to p8) containing overlapping parts of the entire PEDV-MN genome (fragments f1 to f8) were purchased from GenScript, Piscataway, USA. Plasmid p3 is based on pCC1 (Genscript), whereas the remaining plasmids are based on pUC57 (GenScript). Plasmid p1 contains nucleotides (nt) 113832 to 113993 of the vNotI/tk vaccinia virus (5, 62) genome, and these are followed by the T7 promoter and nt 1 to 3,500 of the PEDV strain MN genome. Plasmid p2 contains sequences corresponding to PEDV strain MN nt 3,001 to 7,000. Plasmid p3 contains nt 6,500 to 10,500 of the PEDV strain MN sequence. Plasmids p4 to p7 possess sequences corresponding to nt 10,050 to 13,017, 13,009 to 17,000, 16,501 to 20,500, and 20,000 to 23,999 of the PEDV strain MN genome, respectively. Plasmid p8 contains nt 23,551 to 28,038 of the PEDV strain MN genome, and these are followed by a synthetic poly(A) tail, a NotI cleavage site, and a hepatitis delta ribozyme (HDR) sequence. Fragments derived from plasmids p1 to p8 were subcloned to generate plasmids pA, pB, pC, and pD, which were used to sequentially introduce the full-length PEDV cDNA into the vaccinia virus vNotI/tk via virus-mediated double recombination (Fig. 1). Briefly, fragments f1 and f8, derived from plasmids p1 and p8, were cloned upstream and downstream of the *gpt* (guanosine-phosphoribosyl-transferase) gene in the plasmid pGPT-1 (63), resulting in plasmid pA. Plasmid pB was constructed by introducing fragment f7, derived from plasmid p7, into p2. Plasmid pC was obtained by cloning inserts from p3 and p6 upstream and downstream of the *gpt* gene in pGPT-1. Finally, fragment f5, originating from p5, was subcloned into p4, resulting in pD. The introduction of the full-length PEDV strain MN cDNA into the vNotI/tk vaccinia virus genome was carried out in four rounds of vaccinia virus-mediated homologous recombination, using GPT as a positive and negative selection marker, as described previously (40–42, 44, 63). The plasmid pA was used for vaccinia virus-mediated homologous recombination to generate the *gpt* gene-containing vaccinia recombinant virus recPEDV-MN-1-gpt-8. This virus was selected, using GPT as a positive selection marker. To obtain recPEDV-MN-1-2-7-8 containing nt 1 to 7,000 and 20,000 to 28,038 of the PEDV strain MN genome, recPEDV-MN-1-gpt-8 was used for vaccinia virus-mediated homologous recombination with plasmid pB. The resulting recombinant virus was selected, using GPT as a negative selection marker. The recombination of recPEDV-MN-1-2-7-8 with plasmid pC resulted in recPEDV-MN-1-2-3-gpt-6-7-8 comprising nt 1 to 10,500 and 16,501 to 28,038 of the PEDV strain MN genome. This recombinant virus was obtained after *gpt* positive selection. Finally, to construct recPEDV-MN containing the complete PEDV strain MN cDNA, plasmid pD was used for recombination with recPEDV-MN 1-2-3-gpt-6-7-8, applying GPT as a negative selection marker. The identity of the resulting original PEDV DNA clone (originally recPEDV-MN) was verified via a full-length Sanger sequencing analysis. The generation of recPEDV-MN-S_CV777_-ΔORF3-GFP was carried out in two steps. First, the whole ORF3 gene was replaced by the green fluorescence protein gene (GFP) via two rounds of vaccinia virus-mediated homologous recombination, resulting in recPEDV-MN-ΔORF3-GFP. Second, in this vaccinia virus, the S gene of strain Minnesota (S_MN_) was replaced by the S gene of strain CV777 (S_CV777_) via two rounds of vaccinia virus-mediated homologous recombination.

To generate recombinant vaccinia viruses containing the exact 5′UTR of PEDV strain CV777 (5′UTR_CV777_) instead of the 5′UTR of strain MN (5′UTR_MN_), the 5′UTR_CV777_ was determined by 5′ RACE-PCR (First Choice RLM-RACE Kit, Ambion). In recPEDV-MN-S_CV777_-ΔORF3-GFP, the 5′UTR_MN_ was replaced by the corresponding part of strain CV777 after two rounds of vaccinia virus-mediated homologous recombination, resulting in recPEDV-MN-5′UTR_CV777_-S_CV777_-ΔORF3-GFP. Furthermore, three additional recombinant vaccinia viruses with altered 5′UTR were generated: (i) recPEDV-MN-5′UTR_MN-ΔT48_-S_CV777_-ΔORF3-GFP containing one nucleotide (nt) deletion (T) at position 48 in the 5′UTR_MN_ (ΔT48), (ii) recPEDV-MN-5′UTR_MN-C99T_-S_CV777_-ΔORF3-GFP with the substitution of C with T at nt position 99 (C99T), and (iii) recPEDV-MN-5′UTR_MN-ΔT48, C99T_-S_CV777_-ΔORF3-GFP containing both modifications (ΔT48 and C99T).

The recombinant vaccinia virus recPEDV-MN-5′UTR_CV777_-S_CV777_-ΔORF3-GFP was used to introduce an intact ORF3 gene downstream of the GFP gene via two rounds of vaccinia virus-mediated homologous recombination. The resulting virus was designated recPEDV-MN-5′UTR_CV777_-S_CV777_-GFP and contained, in addition to the parental virus, the last 49 nt of the S gene, including the transcription regulating sequence (TRS), preceding the ORF3 gene.

The latter vaccinia virus served as a starting material for the generation of recPEDV-MN-5′UTR_CV777_-S_MN_-GFP that contained the S gene of strain MN instead of strain CV777. The recPEDV-MN-5′UTR_CV777_-S_MN_-GFP was further modified via vaccinia virus-mediated homologous recombination, resulting in recPEDV-MN-5′UTR_CV777_-S_MN-F226S, L375F, H486P_-GFP and recPEDV-MN-5′UTR_CV777_-S_MN-L375F, H486P_-GFP, respectively. The recPEDV-MN-5′UTR_CV777_-S_MN-L375F, H486P_-GFP contains the S gene of strain MN with two modifications, resulting in a leucine-to-phenylalanine substitution at position 375 and a histidine-to-proline exchange at position 486 (L375F and H486P), whereas recPEDV-MN-5′UTR_CV777_-S_MN-F226S, L375F, H486P_-GFP contains one additional alteration leading to serine instead of phenylalanine at position 226 (F226S). Finally, to generate recPEDV-MN-5′UTR_CV777_-S_MN-L375F, H486P_, the GFP gene was removed from recPEDV-MN-5′UTR_CV777_-S_MN-L375F, H486P_-GFP via two rounds of vaccinia virus-mediated recombination. For the generation of recPEDV-MN-S_CV777_ from the infectious recPEDV-MN synthetic clone, the S_MN_ gene with two modifications (S_MN-L375F, H486P_) was replaced by the S gene of strain CV777. For the generation of recPEDV-MN-ΔpartORF3, a deletion of 49 nucleotides was introduced in the ORF3 of recPEDV-MN that mimics the disrupted ORF3 of the laboratory strain PEDV-CV777. For the generation of recPEDV-MN-5′UTR_CV777_-S_TGEV_ (hereafter named recPEDV-MN-STGEV), the S_CV777_ gene was substituted by the S gene of TGEV strain Purdue (GenBank accession number DQ811789.2). We confirmed the sequence identity of all generated plasmid DNAs and recombinant vaccinia viruses via a full-length Sanger sequencing analysis (Seqlab, Göttingen, Germany), and a summary of all of the generated PEDV clones can be found in Table S1.

### Generation of an inducible cell line expressing the PEDV strain MN N protein.

The cell line BHK-PEDV_MN_-N stably expressing the PEDV-MN nucleocapsid (N) protein is based on BHK-Tet/ON cells that were a kind gift from N. Tautz, University of Lübeck, Germany. Following the transfection of BHK-Tet/ON cells with a plasmid encoding the PEDV strain MN N protein, single colonies were collected after selection with hygromycin (600 μg/mL). A Western blot analysis was performed from cell lysates to determine the expression of the PEDV strain MN N protein 20 h after induction with doxycycline (2 mg/mL).

### Recovery of recombinant PEDV.

For the recovery of all recombinant PEDVs, vaccinia virus DNA was extracted in preparative scale, linearized with NotI (NEB), and used for *in vitro* transcription, as described previously (5, 40–42). The resulting RNA was electroporated into BHK-PEDV_MN_-N cells, as described previously (40–44, 64). After 16 h of incubation, the cells were washed and incubated in serum free minimal essential medium (MEM) containing trypsin (15 μg/mL), which is essential for the cell culture propagation of PEDV (13–15, 17). The cell culture supernatant containing recombinant PEDV was harvested and used for further characterization at 48 h post electroporation. The identity of recombinant PEDVs was determined via a sequencing analysis of the reverse transcriptase (RT)-PCR products that were obtained from the mutated regions.

### Plaque assay.

To determine the titers of the recombinant PEDVs, Vero cells in a 24-well plate were incubated with 200 μL of 10-fold serially diluted virus in the presence of trypsin (80 μg/mL) for 2 h. Subsequently, the inoculum was removed, and the cells were overlaid with 1% carboxymethylcellulose (Sigma-Aldrich) in MEM. At 3 days postinfection (p.i.), plaques were visualized via either GFP expression or indirect immunofluorescence assay.

### Indirect immunofluorescence assay.

PEDV-infected Vero cells in a 24-well plate were fixed with 4% paraformaldehyde and incubated with an N-specific monoclonal antibody (anti-N-PEDV) in phosphate-buffered saline (PBS) containing Triton X-100 (0.1%) and BSA (1%) for 1 h at 37°C. The anti-N-PEDV monoclonal antibody was provided by Mathias Ackermann, Institute of Virology, Vetsuisse Faculty, University of Zurich, Switzerland. After washing, the cells were incubated for 30 min with goat anti-mouse Cy3 secondary antibody, washed, and examined using a fluorescence microscope, as described previously (40).

### Animal studies.

The pathogenicity and virulence of recombinant PEDVs was assessed in newborn specific-pathogen-free (SPF) large white piglets, in compliance with Swiss animal protection law (TSchG SR 455; TSchV SR 455.1; TVV SR 455.163). The experiments were reviewed by the cantonal committee on animal experiments of the canton of Bern, Switzerland and approved by the cantonal veterinary authority (Amt für Landwirtschaft und Natur LANAT, Veterinärdienst VeD Bern, Switzerland) with the authorization number BE125/16. The SPF piglets originated from our breeding facility at the IVI, Mittelhäusern, and are certified free of all epizootic swine diseases, including transmissible gastroenteritis virus, porcine respiratory coronavirus, swine influenza virus A, porcine parvovirus, porcine circovirus type 2, Salmonella suis, Haemophilus parasuis, Lawsonia intracellularis, and Brachyspira hyodysenteriae. Newborn piglets were removed from the sows at the age of 3 to 4 days to guarantee adequate colostrum supply and were kept together in a large group until infection. The piglets were fed with Neopigg Rescuemilk (Cargill, Switzerland) four times daily. The preparation of the milk was carried out according to the manufacturer’s instructions. Drinking water was available *ad libitum*.

Piglets originating from 2 to 3 sows per experiment were assigned randomly to groups of 6 to 9 piglets and were infected orally at the age of 6 and 10 days with a total of 3 mL of viral suspension mixed with Dulbecco’s Modified Eagle Medium (DMEM), using conventional plastic syringes coupled with a flexible rubber tube, thereby enabling proper oral inoculation. The PEDV viruses that were used for infection are described in Table S2. The oral administration of DMEM alone was used as a mock control. The piglets were examined clinically every 24 h, and a clinical scoring sheet was followed to assess liveliness, body shape, walking, breathing, eyes and conjunctives, appetite, and defecation (Table S3). Discontinuation criteria were predefined. In addition, body temperature and body weight were recorded daily, rectal and oronasal swabs were collected daily, and blood samples were collected every two days. Swab samples were incubated in 1 mL of DMEM (containing 1% penicillin/streptomycin, 1% fungizone, and 0.5% bovine serum albumin) for 1 h at 4°C. Blood samples were collected in serum tubes (S-Monovettes, Sarstedt, Nümbrecht, Germany) and left at room temperature for 2 h and at 4°C for 1 h before centrifugation for 10 min at 1,000 × *g*. After the piglets were euthanized, tissue samples were collected for real-time RT-PCR, histopathological analysis, and immunohistochemistry. The samples for the real-time RT-PCR were stored in RINO tubes (Next Advance) containing lysis buffer (30 to 60% guanidinium thiocyanate) and stainless-steel beads (Next Advance) until tissue homogenization was performed by using a Bullet Blender (Next Advance). The tissue samples for the histopathological analysis and immunohistochemistry were stored in 4% buffered formalin. Except for the tissue samples stored in formalin (storage at room temperature), all samples were stored at −70°C.

### Histopathological analysis.

Samples from the small and large intestine, stomach, esophagus, submandibular, mediastinal and mesenterial lymph nodes, tonsils, spleen, thymus, lung, liver, kidney, and pancreas were collected from each piglet upon necropsy and were immediately immersed in 10% neutral buffered formalin. Following fixation, all collected tissues were embedded in paraffin, cut at 4 μm, and stained with hematoxylin and eosin (H&E) for histological evaluation. The length of 30 villi from the jejunum of each piglet was measured by using the CellSens software package (OLYMPUS, Japan).

### Immunohistochemistry.

A 1:10 dilution of the mouse monoclonal antibody mAb204, which is specific for the PEDV membrane (M) protein (anti-M-PEDV) (65, 66), was used for PEDV immunohistochemical detection in the small and large intestine, lung, submandibular, mediastinal and mesenterial lymph nodes, and spleen. This anti-M-PEDV monoclonal antibody was provided by Mathias Ackermann, Institute of Virology, Vetsuisse Faculty, University of Zurich, Switzerland. Paraffin blocks were cut at 3 μm, placed on polysine-coated glass slides, and dewaxed. The slides were then placed in a BOND RX^m^ immunostainer (Leica Biosystems, Germany) and incubated for 30 min with the mAb204 antibody at room temperature. Antigen retrieval was performed by incubating the slides with a citrate buffer for 30 min at 100°C. Then, a Bond Polymer Refine Detection Visualization Kit (Leica Biosystems, Germany) was used for signal detection.

### RNA isolation.

RNA isolation was performed using a KingFisher Flex Purification System and a NucleoMag VET Kit (Macherey-Nagel, no. 744200.4) for the serum and swab samples as well as a NucleoMag RNA Kit (Macherey-Nagel, no. 744350.4) for the tissue samples, respectively, following the manufacturer’s instructions. 10 μL (2 × 10^4^ copies/μL) of enhanced green fluorescent protein (eGFP) RNA were added to each RNA isolation reaction as an internal control to verify the performance of the RNA isolation and real-time RT-PCR, as previously described (67), with modified primers and probe (Table S4).

### Production of an RNA standard from the N-gene of strains MN and CV777.

RNA was isolated from infected cell culture supernatants from the strains CV777 and recPEDV-MN-5′UTR_CV777_-S_CV777_-GFP by using a Qiagen QIAamp Viral RNA Mini Kit (cat. no. 52906), according to the manufacturer’s instructions. The RNA was reverse transcribed with M-MLV reverse transcriptase (Promega, cat. no. M170B) with random primers (Promega, cat. no. C118A) and RNasin Plus (Promega, cat. no. N261A), according to the manufacturer’s instructions. PCR was performed with gene specific primers spanning the whole N-gene (Table S5) by using Phusion High-Fidelity PCR Master Mix with HF Buffer (New England BioLabs) with 5 μL of the RT-reaction mix in a 50 μL end volume of the PCR. The PCR product was cloned into the pET100/d-TOPO cloning vector by using a Champion pET Directional TOPO Expression Kit (Invitrogen), according to the manufacturer´s instructions. From each strain one positive clone was verified via sequencing and used as the template for *in vitro* transcription (Promega RiboMAX Large Scale RNA Production Systems; cat. no. P1300) after linearization with ClaI (New England BioLabs). The RNA was purified by using a Macherey-Nagel NucleoSpin RNA Kit, according to the manufacturer´s instructions, and it was quantified by using a NanoDrop ND-1000 spectrophotometer (Witec AG, Switzerland). Then, the RNA was diluted to a concentration of 1 μg/μL and stored in aliquots at −80°C until use.

### Quantitative RT-PCR.

TaqMan Fast Virus 1-Step Master Mix (Applied Biosystems) was used, according to the manufacturer’s instructions. Specific primers and probes were added in a multiplex reaction for PEDV and the internal control RNA eGFP (Table S4). In each run, 2 μL of a dilution series of PEDV RNA, as described previously, ranging from 2 × 10^1^ to 2 × 10^7^ copies per reaction was added to calculate the genome equivalent (GE) per milliliter of serum or per milligram of tissue of the analyzed samples.

### Amplicon preparation for deep sequencing from contact piglet 2 from the PEDV/TGEV chimeric virus experiment.

The RNA of a jejunum sample from contact piglet 2 was isolated by using a NucleoMag RNA Kit (Macherey-Nagel, no. 744350.4), according to the manufacturer’s instructions. Subsequently, the amount of extracted RNA was determined prior to reverse transcription. cDNA was synthesized using a three step protocol M-MLV kit (Invitrogen) that was established in the lab. To maximize the coverage depth and to perform deep sequencing for each virus sample, we generated 13 overlapping amplicons of approximately 2,500 to 2,700 bp in length that overlapped the adjacent amplicons by 300 to 500bp (Table S6). PCR amplification was performed for each amplicon by using CloneAmp HiFi PCR Premix (TaKaRa, no. 639298) to minimize errors during PCR. The amplified DNA was purified using NucleoSpin Gel and a PCR Clean-up Kit (Macherey-Nagel, no. 740609.250), following the manufacturer’s recommendations. Amplicon concentrations were determined via spectrophotometry as well as via a Qubit fluorometric quantification system and a dsDNA High Sensitivity Assay Kit from Invitrogen (no. Q32854). The final concentration was 30 to 100 ng/μL.

### Library preparation, Illumina sequencing, and data analysis from contact piglet 2 from the PEDV/TGEV chimeric virus experiment.

Prior to sequencing, all amplicons were equimolarly pooled to a final concentration of 5 ng/μL. Paired-end libraries were prepared by using a Nextera Library XT DNA Library Kit (Illumina) and were analyzed by using an Agilent fragment analyzer. Samples were sequenced at the UZH Zürich (2 × 300bp reads). The overall read quality was assessed by using FastQC (version 0.11.7), and the Trimmomatic software package (version 0.36) was used to remove adapters and any low-quality reads (≤Q20) from the samples. Bowtie2 (version 2.3.4.1) was then used to map reads to the PEDV-MN-5′UTR_CV777_-S_TGEV_ sequence, and mutations were called using the LoFreq variant-caller software package (version 2.0). Variant annotation and effect prediction were performed using SnpEff (version 4.0), and called variants were further visualized in R (version 3.6.1). Variants with an allele frequency of less than 10% were excluded from all subsequent analyses. SAMtools (version 1.8) was used to generate a consensus sequence from the sample.

### Statistical evaluation.

The results showing clinical scores and viral RNA titers were statistically analyzed by using the GraphPad Prism (Version 5.03, San Diego, CA, USA) software package. The variables among groups were compared using one-way analyses of variance (ANOVAs) that were followed by Tukey’s tests. The length of the intestinal villi from each group was statistically compared by using either multilevel linear regression models or nonparametric tests, such as the Kruskal-Wallis H test from the statistical package R (R Core Team, USA). A *P* value of <0.05 was considered to be indicative of a statistically significant result in all cases.

### Data availability.

Calculations were performed on UBELIX (http://www.id.unibe.ch/hpc), the HPC cluster at the University of Bern. The sequencing data from this experiment have been deposited into the Sequence Read Archive (SRA) from NCBI and with the BioProject ID PRJNA785784.
