# Supplementary figures and images for: Reverse Genetic Assessment of the Roles Played by the Spike Protein and ORF3 in Porcine Epidemic Diarrhea Virus Pathogenicity
Source: J Virol. 2023 Jun 26;97(7):e01964-22. doi: 10.1128/jvi.01964-22 (PMC10373562; doi:10.1128/jvi.01964-22)

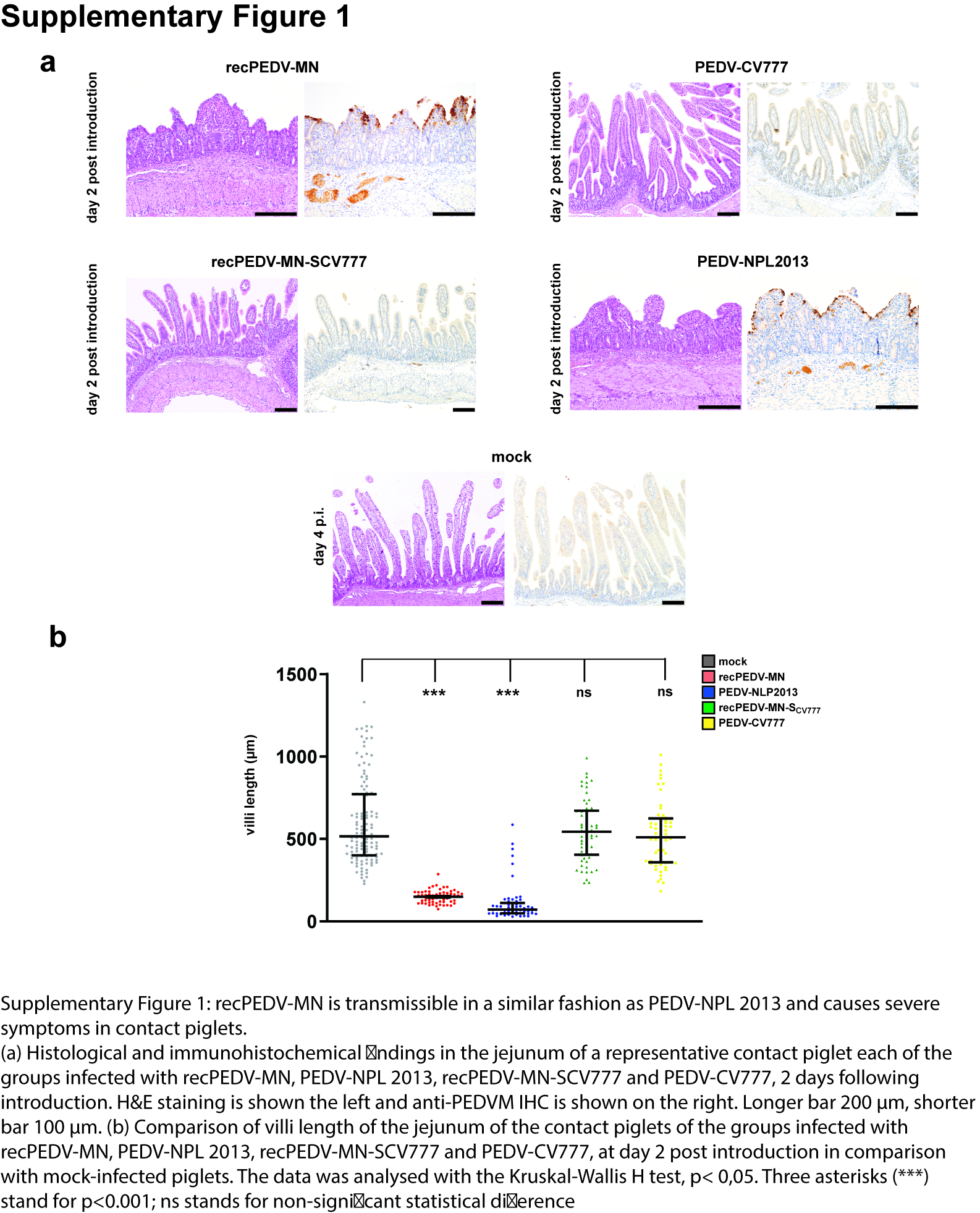

Supplement: Supplemental file 1 — Fig. S1. Download jvi.01964-22-s0001.tif, TIF file, 14.4 MB [file jvi.01964-22-s0001.tif]

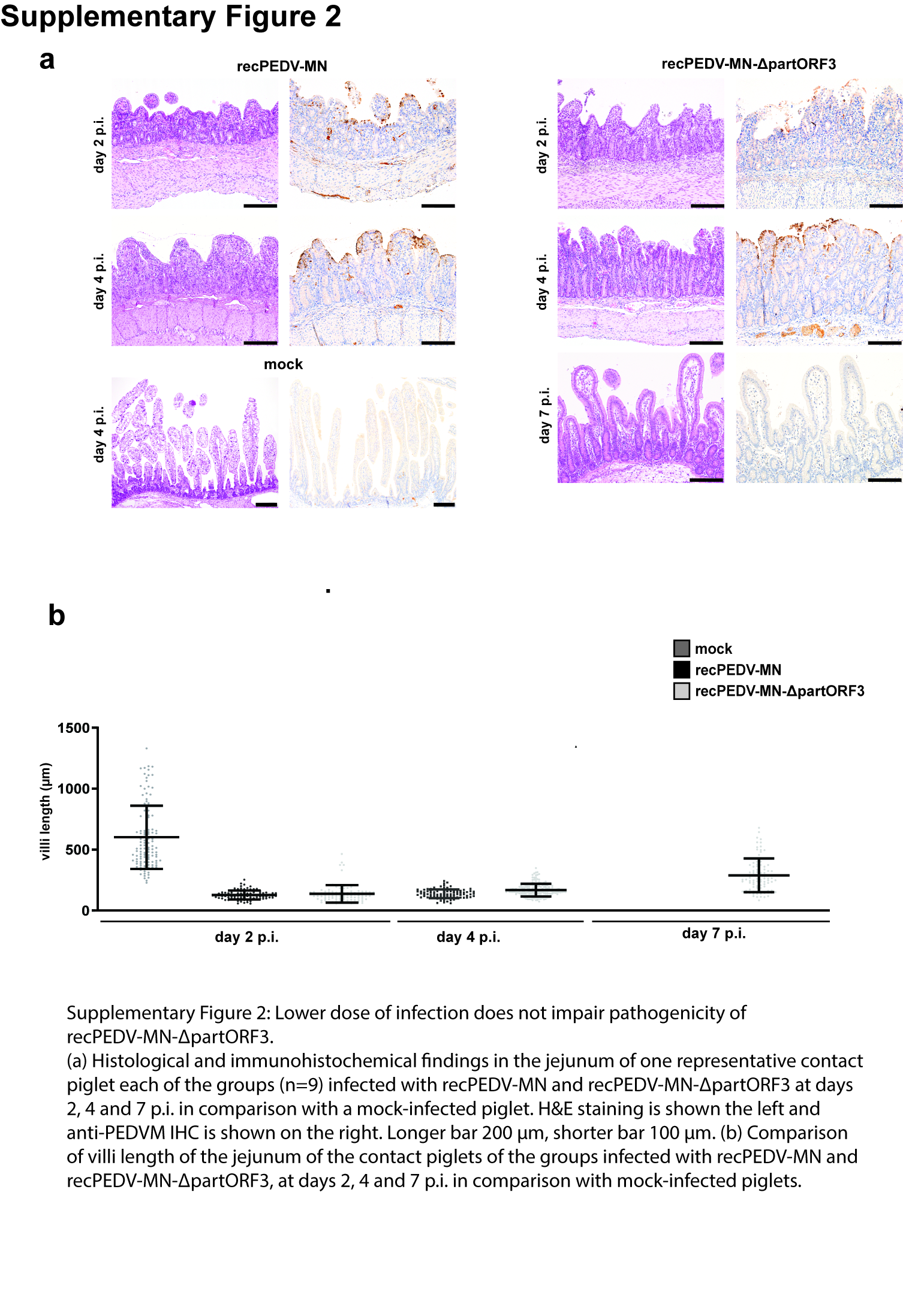

Supplement: Supplemental file 2 — Fig. S2. Download jvi.01964-22-s0002.tif, TIF file, 14.9 MB [file jvi.01964-22-s0002.tif]
