# Supplementary material for: Reverse Genetic Assessment of the Roles Played by the Spike Protein and ORF3 in Porcine Epidemic Diarrhea Virus Pathogenicity
Source: J Virol. 2023 Jun 26;97(7):e01964-22. doi: 10.1128/jvi.01964-22 (PMC10373562; doi:10.1128/jvi.01964-22)
Supplement: Supplemental file 3 — Tables S1 to S6. Download jvi.01964-22-s0004.docx, DOCX file, 0.02 MB [file jvi.01964-22-s0004.docx]

**Supplementary Tables:**

**Table S1.** Summary of all recombinant PEDV clones generated within the scope of this study. The underlined clones were used for the in vivo experiments.

| **Recombinant PEDV strain** | **Description** |
| --- | --- |
| Original rec PEDV-MN | Original clone generated from the wild-type PEDV Minnesota (MN) strain (Genbank acession nr. KF468752) via vaccinia virus reverse genetics |
| recPEDV-MN | Clone generated from the wild-type PEDV Minnesota (MN) strain sequence (Genbank acession nr. KF468752) with four modifications:  -Two changes in the 5’-UTR, namely a deletion of U48 (∆U48) and the replacement of C99 by U  - Two changes in the S gene sequence: a phenylalanine at aa position 375 and proline at aa position 486. Genbank acession nr. OL704806. |
| recPEDV-MN-∆ORF3-GFP | recPEDV-MN with replacement of ORF3 by GFP |
| recPEDV-MN-S_CV777_-∆ORF3-GFP | recPEDV-MN with the S gene from the PEDV CV777 strain; replacement of the ORF3 gene by GFP |
| recPEDV-MN-5’UTR_CV777_-S_CV777_-∆ORF3-GFP | recPEDV-MN with 5’UTR and the S gene from the PEDV CV777 strain; replacement of the ORF3 gene by GFP |
| recPEDV-MN-5’UTR_MN-∆T48_-S_CV777_-∆ORF3-GFP | recPEDV-MN with 5’UTR displaying one nt deletion (T) at position 48 (∆T48) and the S gene from the PEDV CV777 strain; replacement of ORF3 by GFP |
| recPEDV-MN-5’UTR_MN-C99T_-S_CV777_-∆ORF3-GFP | recPEDV-MN with 5’UTR displaying two modifications: a substitution of C with T at nt position 99 (C99T) and the S from the PEDV CV777 strain; replacement of ORF3 by GFP |
| recPEDV-MN-5’UTR_MN-∆T48, C99T_-S_CV777_-∆ORF3-GFP | recPEDV-MN with 5’UTR displaying two modifications: one nt deletion (T) at position 48 (∆T48) and a substitution of C with T at nt position 99 (C99T); the S gene from the PEDV CV777 strain; replacement of ORF3 by GFP |
| recPEDV-MN-5’UTR_CV777_-S_CV777_-GFP | recPEDV-MN with 5’UTR and the S gene from the PEDV CV777 strain; insertion of the last 49 nt of the S gene, including TRS preceding ORF3; intact ORF3 gene insertion downstream of the GFP |
| recPEDV-MN-5’UTR_CV777_-S_MN_-GFP | recPEDV-MN with 5’UTR from the PEDV CV777 strain and the S from the PEDV MN strain; insertion of the last 49 nt of the S gene, including TRS preceding ORF3; intact ORF3 gene insertion downstream of the GFP |
| recPEDV-MN-5’UTR_CV777_-S_MN-L375F, H486P_-GFP | recPEDV-MN with 5’UTR from the PEDV CV777 strains; S from the PEDV MN strain with two modifications: leucine (L) to phenylalanine (F) substitution at position 375 (L375F), and a histidine (H) to proline (P) exchange at position 486 (H486P); insertion of the last 49 nt of the S gene, including TRS preceding ORF3; intact ORF3 gene insertion downstream of the GFP |
| recPEDV-MN-5’UTR_CV777_-S_MN-F226S, L375F, H486P_-GFP | recPEDV-MN with 5’UTR from the PEDV CV777 strain and the last 49 nt of the S gene, including TRS of the ORF3 gene; S gene with three modifications: phenylalanine (F) to serine (S) substitution at position 226 (F226S), leucine (L) to phenylalanine (F) substitution at position 375 (L375F), and a histidine (H) to proline (P) exchange at position 486 (H486P); ORF3 replaced by GFP |
| recPEDV-MN-5’UTR_CV777_-S_MN-L375F, H486P_-GFP | recPEDV-MN with 5’UTR from the PEDV CV777 strain and the last 49 nucleotides of the S gene, including TRS of the ORF3 gene; S gene with two modifications: leucine (L) to phenylalanine (F) substitution at position 375 (L375F), and a histidine (H) to proline (P) exchange at position 486 (H486P); insertion of the last 49 nt of the S gene, including TRS preceding ORF3; intact ORF3 gene insertion downstream of the GFP |
| recPEDV-MN-S_CV777_ | recPEDV-MN with 5’UTR displaying two modifications: one nt deletion (T) at position 48 (∆T48) and substitution of C with T at nt position 99 (C99T); S gene from the PEDV CV777 strain |
| recPEDV-MN-ΔpartORF3 | recPEDV-MN with 5’UTR displaying two modifications: one nt deletion (T) at position 48 (∆T48), a substitution of C with T at nt position 99 (C99T); deletion of 49 nts in the ORF3 gene |
| recPEDV-MN-5’UTR_CV777_-S_TGEV_/ recPEDV-MN-STGEV | recPEDV-MN with 5'UTR from CV777 and S gene of TGEV strain Purdue (GenBank accession nr. DQ811789.2) |

*Abbreviations:aa: aminoacid; nt: nucleotide.*

**Table S2.** PEDV viruses used for animal studies in piglets.

|  |  |
| --- | --- |
|  |  |
| **PEDV strain**  **(Isolates and recombinants)** | **GenBank Accession number** |
|  |  |
|  |  |
| CV777 | AF353511 |
|  |  |
| PEDV-NPL 2013 | KM052365 |
|  |  |
| PEDV-MN | KF468752 |
|  |  |
| recPEDV-MN | OL704806 (this study) |
|  |  |
| recPEDV-MN-S_CV777_ | Will be submitted |
|  |  |
| recPEDV-MN-ΔpartORF3 | Will be submitted |
|  |  |
| recPEDV-MN-S_TGEV_ | Will be submited |
|  |  |
|  |  |

**Table S3:** Parameters used for clinical score determination during *in vivo* experimental PEDV infections in piglets.

|  |  |  |
| --- | --- | --- |
| **Parameter** | **Criteria** | **Score** |
|  |  |  |
| **1. Liveliness** | Attentive, curious, stands up immediately | 0 |
|  | Slightly reduced, stands up hesitantly but without help | 1 |
|  | Clearly reduced, gets up only when forced to and lies down again | 2 |
|  | Strongly reduced, somnolent for more than 24 h | **3** |
|  |  |  |
| **2. Body shape** | Full stomach, “round” body | 0 |
|  | Empty stomach | 1 |
|  | Empty stomach, sunken flanks | 2 |
|  | Empty stomach, sunken flanks, thinned body muscles | **3** |
|  |  |  |
| **3. Walking** | Well-coordinated movements, relaxed straight back | 0 |
|  | Stiffness when standing up, hesitant walking | 1 |
|  | Constantly bent back, stiff walking | 2 |
|  | Unable to walk due to weakness or lameness for more than 24 h | **3** |
|  |  |  |
| **4. Breathing** | Normal, barely visible chest movement | 0 |
| (judge before | Slightly elevated frequency and chest movements | 1 |
| approaching pig) | Clearly elevated breathing frequency and chest movement | 2 |
|  | Breathing through open mouth | **3** |
|  |  |  |
| **5. Eyes /** | Normal eye tension with light pink conjunctiva | 0 |
| **conjunctiva** | Decreased eye tension, pale or reddened conjunctiva | 1 |
|  | Sunken eyes, pale or reddened conjunctiva, mild dehydration | 2 |
|  | Severely sunken eyes, pale grey conjunctiva, severe dehydration | **3** |
|  |  |  |
| **6. Appetite** | Greedy, hungry | 0 |
|  | Drinks, eats slowly when fed | 1 |
|  | Doesn't drink/eat when fed, but sniffs milk/food | 2 |
|  | Doesn't drink/eat at all, no interest in milk /food for more than 24 h | **3** |
|  |  |  |
| **7. Defecation** | Soft faeces, normal amount | 0 |
|  | Mild diarrhoea | 1 |
|  | Severe diarrhoea | 2 |
|  | Watery and/or bloody diarrhea | **3** |
|  |  |  |

**Monitoring:** After infection and on all days with clinical symptoms, examination of the pigs is performed at least once a day every day by a veterinarian (twice a day if criteria for discontinuation may be reached before the next day). When symptoms occur, monitoring is performed by the same person whenever possible to ensure unbiased clinical assessment.

**Criteria for discontinuation of the experiment:**

A pig will be euthanized immediately if it reaches:

- A total clinical score of 14 and higher for all parameters together

OR

- A score of 3 for at least one of the parameters #1 and 3 to 6.

**Table S4:** Primers and probes used for quantitative RT-PCR multiplex reaction for PEDV detection.

|  |  |  |  |
| --- | --- | --- | --- |
|  |  |  |  |
| **Type** | **Sequence (5'-3')** | **Bp** | **Genome Position^a^** |
|  |  |  |  |
|  |  |  |  |
| **PEDV forward primer** | GAACAACCTTCCAATTGGCATTT | 23 | 26575 - 26597 |
|  |  |  |  |
| **PEDV reverse primer** | TAGTGGGTTCAGTCTTTGCGC | 21 | 26678 - 26698 |
|  |  |  |  |
| **PEDV probe** | FAM-GACTCGTACTGAGGGTGTT-MGBMFQ | 19 | 26640 - 26658 |
| **MGB** |  |  |  |
|  |  |  |  |
| **eGFP forward primer** | GGGCACAAGCTGGAGTACAAC | 21 | 511 - 531 |
|  |  |  |  |
| **eGFP reverse primer** | CACCTTGATGCCGTTCTTCTG | 21 | 568 - 588 |
|  |  |  |  |
| **eGFP probe** | YYE-ACAACAGCCACAACGTCTATATCATGGCC-BHQ1 | 29 | 533 - 561 |
|  |  |  |  |
|  |  |  |  |

*Abbreviations: Bp: base pairs; MGBNFQ: minor groove binder nonfluorescent quencher*

*^a^ Genome position according to PEDV strain CV777 (Accession Number AF353511) and eGFP (Accession Number U55761), respectively.*

**Table S5:** Gene specific primers spanning the whole N-gene used for production of a RNA standard from the N-gene of strains MN and CV777.

| **Type** | **Sequence (5'-3')** | | **Bp** | **Genome Position** |
| --- | --- | --- | --- | --- |
|  |  | |  |  |
| **PEDV CV777 N- gene forward primer** | CACCATGGCTTCTGT CAGCTTTCAGG^c^ | 26 | | 26374 - 26395^a^ |
|  |  | |  |  |
| **PEDV-MN N- gene forward primer** | CACCATGGCTTCTGTCAGTTTTCAGGAT^c^ | | 28 | 26379 - 26402^b^ |
|  |  | |  |  |
| **PEDV CV777 N- gene reverse primer** | TTAATTTCCTGTATC GAAGATCTCGTT | | 27 | 27699 - 27673^a^ |
|  |  | |  |  |
| **PEDV-MN N-gene reverse primer** | TTAATTTCCTGTGTCGAAGATCTCG | | 25 | 27704 - 27680^b^ |
|  |  | |  |  |

*Abbreviation: Bp: base pairs*
*a Genome position according to PEDV strain CV777 (Accession Number AF353511)*
*b Genome position according to PEDV strain Minnesota (Accession Number KF468752)*

*c CACC overhang used for direct cloning into pET TOPO® vectors*

**Table S6:** Primers used for amplicon generation from viral RNA recovered from the jejunum of contact piglet 2.

|  |  |  |  | |
| --- | --- | --- | --- | --- |
|  |  |  | |  |
| **Primer Name** | **Sequence (5'-3')** | **Bp** | **Accession** | |
|  |  |  |  | |
|  |  |  |  | |
| **PEDVwg-F 1** | AGATTTTCTATCTACGGATAGTTAGCTCTTTTT | 33 | *KF468752* | |
| **PEDVwg-F 2** | ACAACACTCCTTATAAAACTTACAGCTGC | 29 | *KF468752* | |
| **PEDVwg-F 3** | GAGGCATTAGGTCTTAAGGTCTTTAATGT | 29 | *KF468752* | |
| **PEDVwg-F 4** | CACCTTAGAGACCCATTAATTGGTAAT | 27 | *KF468752* | |
| **PEDVwg-F 5** | GTAACACAGAACACACTTGGCATGT | 25 | *KF468752* | |
| **PEDVwg-F 6** | TGGTATTGGTGGTGAGCGG | 19 | *KF468752* | |
| **PEDVwg-F 7** | TGGTGTTGTCACATTAGATAATCAGGAT | 28 | *KF468752* | |
| **PEDVwg-F 8** | TGTTGTTTGTGGCTCTCAAACTGT | 24 | *KF468752* | |
| **PEDVwg-F 9** | CGCACACCTTTTGCCTTTACTTA | 23 | *KF468752* | |
| **PEDVwg-F 10** | GCCTTCTATTTACAAGATACAACGTATGTG | 30 | *KF468752* | |
| **PEDVwg-F 11** | GTAACTGATGGACCACGGTACT | 22 | *DQ811789* | |
| **PEDVwg-F 12** | CCCAGAACTATGTATCAGCCTAGAG | 25 | *DQ811789* | |
| **PEDVwg-F 13** | ATCGTGGCCGCAAACG | 16 | *KF468752* | |
| **PEDVwg-R 1** | ATCCCACATTGTATTAAGACCAGTGAT | 27 | *KF468752* | |
| **PEDVwg-R 2** | GTTGAACATCTTCCTGGACAGGA | 23 | *KF468752* | |
| **PEDVwg-R 3** | TGGACAGGAACGCGCTTAAG | 20 | *KF468752* | |
| **PEDVwg-R 4** | GACGCAGTTTTTCAGTTGAGATAGAGT | 27 | *KF468752* | |
| **PEDVwg-R 5** | CATAAGTAGATGCAACACTCTGCAAC | 26 | *KF468752* | |
| **PEDVwg-R 6** | AGTTGGCACAGTGAACTATGCACT | 24 | *KF468752* | |
| **PEDVwg-R 7** | CGGATGTAGCAATGCGATTAAAG | 23 | *KF468752* | |
| **PEDVwg-R 8** | CCTTGTATCCCCACTGCTGTATATC | 25 | *KF468752* | |
| **PEDVwg-R 9** | TGACAGGTATAAGGTAGAGCAATCTCC | 27 | *KF468752* | |
| **PEDVwg-R 10** | ATGTTGAGGCTATGGGTTGACC | 22 | *DQ811789* | |
| **PEDVwg-R 11** | TGTCAACTCAGGTACAGTCCAATT | 24 | *DQ811789* | |
| **PEDVwg-R 12** | AGTGGGTTCAGTCTTTGCG | 19 | *KF468752* | |
| **PEDVwg-R 13** | CATATCAACACCGTCAGGTCTTCA | 24 | *KF468752* | |
|  |  |  |  | |
