# Supplementary material for: Reverse Genetic Assessment of the Roles Played by the Spike Protein and ORF3 in Porcine Epidemic Diarrhea Virus Pathogenicity
Source: J Virol. 2023 Jun 26;97(7):e01964-22. doi: 10.1128/jvi.01964-22 (PMC10373562; doi:10.1128/jvi.01964-22)
Supplement: Supplemental file 4 — Legends of Fig. S1 and S2. Download jvi.01964-22-s0003.docx, DOCX file, 0.01 MB [file jvi.01964-22-s0003.docx]

**Supplementary figure legends:**

**Supplementary Figure 1: recPEDV-MN is transmissible in a similar fashion as PEDV-NPL 2013 and causes severe symptoms in contact piglets.**

**(a)** Histological and immunohistochemical findings in the jejunum of a representative contact piglet each of the groups infected with recPEDV-MN, PEDV-NPL 2013, recPEDV-MN-S_CV777_ and PEDV-CV777, 2 days following introduction. H&E staining is shown the left and anti-PEDV_M_ IHC is shown on the right. Longer bar 200 µm, shorter bar 100 µm. **(b)** Comparison of villi length of the jejunum of the contact piglets of the groups infected with recPEDV-MN, PEDV-NPL 2013, recPEDV-MN-S_CV777_ and PEDV-CV777, at day 2 post introduction in comparison with mock-infected piglets. The data was analysed with the Kruskal-Wallis H test, p< 0,05. Three asterisks (***) stand for p<0.001; ns stands for non-significant statistical difference.

**Supplementary Figure 2: Lower dose of infection does not impair pathogenicity of recPEDV-MN-ΔpartORF3.**

**(a)** Histological and immunohistochemical findings in the jejunum of one representative contact piglet each of the groups (n=9) infected with recPEDV-MN and recPEDV-MN-ΔpartORF3 at days 2, 4 and 7 p.i. in comparison with a mock-infected piglet. H&E staining is shown the left and anti-PEDV_M_ IHC is shown on the right. Longer bar 200 µm, shorter bar 100 µm. **(b)** Comparison of villi length of the jejunum of the contact piglets of the groups infected with recPEDV-MN and recPEDV-MN-ΔpartORF3, at days 2, 4 and 7 p.i. in comparison with mock-infected piglets.
